# “Ecce Homo” by Antonello da Messina, from non-invasive investigations to data fusion and dissemination

**DOI:** 10.1038/s41598-021-95212-2

**Published:** 2021-08-05

**Authors:** Fauzia Albertin, Chiara Ruberto, Costanza Cucci, Marco Callieri, Marco Potenziani, Eliana Siotto, Paolo Pingi, Roberto Scopigno, Matteo Bettuzzi, Rosa Brancaccio, Maria Pia Morigi, Lisa Castelli, Francesco Taccetti, Marcello Picollo, Lorenzo Stefani, Francesca de Vita

**Affiliations:** 1 Enrico Fermi Historical Museum of Physics and Study and Research Center, 00184 Rome, Italy; 2 INFN - National Institute of Nuclear Physics, 40126 Bologna, Italy; 3 Department of Physics and Astronomy “Augusto Righi”, University of Bologna, 40126 Bologna, Italy; 4 INFN - National Institute of Nuclear Physics, 50019 Sesto Fiorentino, Florence, Italy; 5 Department of Physics and Astronomy, University of Florence, 50019 Sesto Fiorentino, Florence, Italy; 6 CNR - IFAC - National Research Council, Institute of Applied Physics “Nello Carrara”, 50019 Sesto Fiorentino, Florence, Italy; 7 CNR - ISTI - National Research Council, Institute of Information Science and Technologies “Alessandro Faedo”, 56124 Pisa, Italy; 8 ALEF Conservation and Restoration Company, 43121 Parma, Italy

**Keywords:** Characterization and analytical techniques, Imaging techniques, Characterization and analytical techniques, Imaging techniques, Optical spectroscopy, Infrared spectroscopy, Near-infrared spectroscopy, Spectrophotometry, Optical spectroscopy, Infrared spectroscopy, Near-infrared spectroscopy, Spectrophotometry, Software, Information technology, Computer science, Scientific data

## Abstract

Scientific investigations of artworks are crucial in terms of preservation since they provide a measurable evaluation of the materials and the state of conservation. This is the case of Antonello da Messina’s painting “Ecce Homo”: its delicate state of conservation, with the need for constant monitoring, required a broad and in-depth diagnostic campaign to support the restorers. The project was carried out entirely in situ using non-invasive cutting-edge techniques and proposes a multimodal and data-centric approach, integrating 3D and 2D methodologies. The surface irregularities and the support were analysed with a structured-light 3D scanner and X-ray tomography. The painting materials were investigated with X-ray fluorescence scanning (MA-XRF) and reflectance hyperspectral imaging (HSI). Primarily, the data were jointly used for a scientific scope and provided new knowledge of the painting in terms of materials and painting techniques. In addition, two web-based interactive platforms were developed: one to provide restorers and experts with a new perspective of the hidden geometries of the painting, and the other targeted at the general public for dissemination purposes. The results of the Ecce Homo scientific analysis were exhibited, using a touch-screen interface, and developed for different user levels, from adults to kids.

## Introduction

Antonello da Messina (ca. 1430–1479), one of the most important and internationally renowned Italian painters, was recognized by Giorgio Vasari as the artist who introduced oil painting to Italy^1^ during the early Italian Renaissance, and influenced many Italian artists, especially in Venice^1^. His most famous masterpieces are currently part of several international collections. “Saint Jerome in His Study” (ca. 1475) and “Salvator Mundi” (1476) are in collections at the National Gallery, London (UK)^2^, “Portrait of a Man” (around 1470) is in the Metropolitan Museum of Art, New York (US)^3^, and the portrait of a man, known as “The Condottiero” (1475) is in the Musèe du Louvre, Paris (France)^4^. Much of his artistic production, however, remains in his homeland, such as the masterpiece “The Virgin Annunciate” (1476) in the Abatellis Palace in Palermo, “The Annunciation” (1474) in the Bellomo Palace Regional Gallery in Syracuse, and “Portrait of a Man” in the Mandralisca Museum in Cefalù (Palermo).

**Figure 1:**
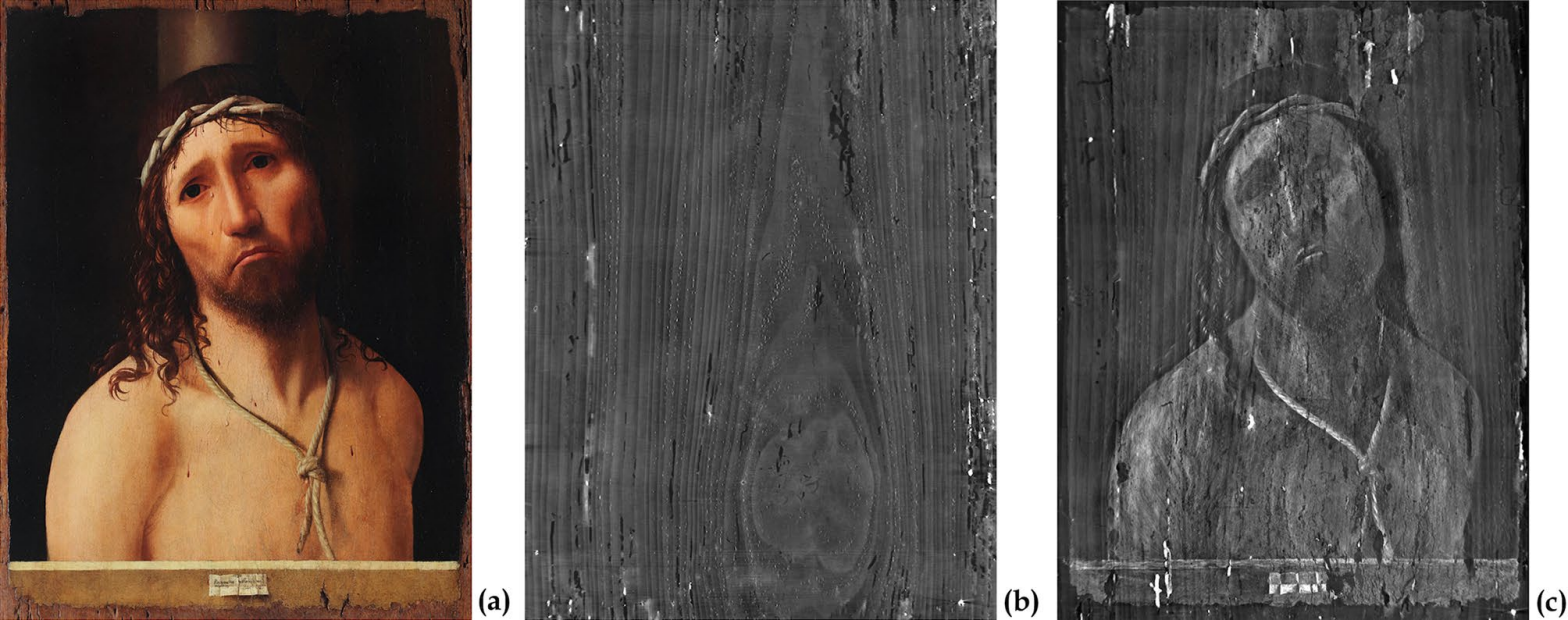
The Ecce Homo painting and its tomographic investigation. (**a**) Colorimetric calibrated image reconstructed from hyperspectral imaging (HSI) visible data. (**b**) Section of the tomographic volume related to the middle part of the board. (**c**) Sections of the tomographic volume related to the pictorial layers.

The “Ecce Homo” presented here is part of the Collegio Alberoni Collection in Piacenza (Italy). This artwork is one of the five known versions of the "Christ at the column" and, undoubtedly, is one of the world’s most recognized masterpieces of the artist. The painting (Fig. 1a) is an oil painting on wood (38.5 × 48.5 × 0.5 cm). According to scholars, this painting can be dated from 1475 onwards, when the Sicilian artist was recorded as being in Venice. Ecce Homo belongs to this artistic milieu in terms of his working techniques and style. The finely painted figure and innovative posture of Christ, represented in a frontal position and looking towards the observers, expresses great suffering. His pain is superbly conveyed to the observer by for example his eyebrows, lips turned downwards, and shining tears. On the other hand, the background is dark and devoid of detail. As with other Antonello da Messina paintings, a cartouche containing the painter’s signature and the date of its execution is depicted at the bottom of the image, over a parapet.

Over time the painting has undergone many, and not always respectful, interventions. The first documented one dates back to the early 1900s when the natural warp of the support was rectified. At that time, the support was moistened, significantly planed down, and then pressed to lie flat^5^. Consequently, the paint layer was forced to return to the initial flat dimension, leading to an overlap of some areas and the collapse of others into the holes caused by the xylophagous insects that had infested the support of the painting. Moreover, to avoid future warping of the board and allowing only small movements, a wooden cradle with sliding elements was glued on the reverse of the panel^5^. Afterwards, a massive cleaning was later followed by a complete re-painting to obtain a new patina.

In the mid-twentieth century, the painting surface was cleaned twice more, and the lacunae and missing areas were filled and integrated. The cradle, improperly positioned at the beginning of the century and resulting in an undulation of the structure, was then removed, and replaced with a new one, currently visible on the reverse side of the painting^5^. All these alterations have left the painting in a delicate conservation state, needing constant monitoring and requiring careful management of the environment inside a special microclimate case, where the panel is kept.

In order to carry out a scientific evaluation of the conservation state of the painting and an analysis of its materials, a multimodal non-invasive investigation campaign was carried out with the close collaboration of four research groups and the restorer of the artwork.

The “ECCEHOMO” project^6^ aimed to combine several cutting-edge methods for a systematic analysis of the artwork through non-invasive techniques, from the high-definition 3D map of its superficial and inner layers to 2D imaging techniques, which provide the chemical mapping of materials. Given the fragility of the painting and the possible risk of transportation, all the investigations were performed in situ*.*

In order to non-invasively analyse the geometry of the pictorial surface and its irregularities, and to better evaluate the flaking of the pictorial layers, a structured-light 3D metrological survey was used to digitize the external surface of the artwork. To visualize and study the conditions of the support, the extent of the xylophage’ attacks, and the preservation state of the pictorial layers, an X-ray tomographic analysis was performed. The information obtained on the distribution of the damage from woodworm and the pictorial layer flaking was combined to create a global map of the critical elements of the painting, a fundamental guide in the evaluation of any conservation intervention.

To analyse the painting materials, the choice of pigments, and the artist’s technique by non-invasively examining the pictorial layers, two highly complementary imaging techniques, X-ray fluorescence scanning (MA-XRF) and hyperspectral imaging (HSI) in the visible and near and short-wave infrared range (Vis–NIR-SWIR), were combined. In addition, to document the state of conservation of the upper varnish layers and to complement the other imaging techniques, UV-fluorescence imaging was carried out.

Considering the importance of the artwork and its fragility, non-destructive and non-invasive techniques had to be available in situ. In addition, as the stability of the environmental conditions of the painting were a priority, all the analyses were carried out on the artwork in the same exhibition hall, taking care to stabilize the temperature and the humidity.

All the analyses were only carried out using instruments specifically designed for artwork investigations, i.e. that minimized any impact on the object and were easily adaptable to the specific working conditions.

In order to introduce novel paradigms for the effective exploitation of the datasets gathered, this project studied how to combine data for providing better answers to specific conservation needs, but also proposed how to re-use them for different purposes. With this aim, the data publishing scenario was explored, exploiting the multimodal datasets as an informative layer for building two applications: a web platform, intended for technical users, and a museum kiosk, addressed to a general audience.

In addition to being a specific case study, this complex investigation and the methodology used to integrate and exploit all the results—from 3 to 2D imaging data—provide guidelines for an innovative model for documenting paintings.

## Results and discussion

### Behind the painting: the key role of the support

High-resolution 3D acquisition and X-ray tomographic analysis were used to investigate all of the painting support from its surface to the inner layers. The combined use of these two non-invasive techniques resulted in a morphological map of the whole painting, which enhanced the knowledge of the artwork’s history, and also provided a reference for future intervention choices.

#### The support over time

Although Antonello da Messina pursued the ideal of technical perfection in his painting, he nevertheless chose a wooden board with a large knot in the centre (Fig. 1b). The knot, revealed by X-ray tomography and located behind the heart of the Christ figure, could be identified as a possible cause of instability of the pictorial layers and of some of the detachments and lacunae.

In addition, the tomographic investigation and the examination of the sub-volume related only to the painting layers, revealed numerous restored lacunae, mainly oriented in the same direction as the wood fibres, and several superficial woodworm tunnels (Fig. 1c).

Today the Ecce Homo does not have a frame. An examination of the borders of the painting revealed traces that confirm the previous presence of a frame: several remains of a thin film of animal glue, probably used for the frame adhesion, can still be seen where the borders of the paint film are undamaged. Several clues suggest that the frame had been fixed on the surface after laying down the preparatory layers. First of all, the paint film has almost a rectangle shape and the straight borders suggest that the colour was applied against a frame structure that masked the outer part of the board. The top and bottom sides are more damaged, which is compatible with board warping that ended up detaching the frame, damaging the adjacent paint layers. Finally, the left and right sides are still almost straight, possibly because the vertical parts of the frame were aligned with the grain of the board, resulting in less warping and cracking of the adjacent paint film.

**Figure 2:**
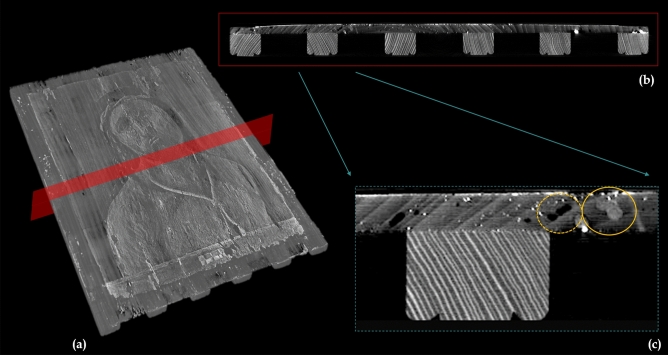
Tomographic analysis of the painting support. (**a**) 3D rendering of the entire tomographic volume and the virtual plane analysed (in red). (**b**) The corresponding tomographic slice shows the massive cradling, slim support, and extremely thin painting layers. (**c**) A detail of the previous section where several woodworm tunnels are visible, some are *empty,* and some have been *filled* with dust and wood residues (highlighted with dashed and continuous yellow circles).

The tomographic investigation (Fig. 2a) confirmed the thinness of the support, most probably caused by the heavy planing carried out in the intervention in the early 1900s. The panel is about 7.5 mm thick in the central area and approximately 6 mm near the sides (Fig. 2b). Similarly, the painting layers are exceptionally thin, just 0.2—0.4 mm. Despite the thinness of the board, damage from woodworm appears to be severe and extended. As shown in Fig. 2c, the tomography shows different series of woodworm tunnels, with diameters in the range of 0.3–0.6 mm and 1.5–2 mm, respectively, which are presumably related to separate attacks. There are currently no active infestations.

Fortunately, the area behind the central part of the painting seems less affected, thus not compromising the stability of the paint film in the focal area of the portrait. Some tunnels, mostly in the peripheral area, appear to have been partially filled and restored.

Integrating the tomographic volume data with the 3D surface model, produced a clear map of the extent of the woodworm tunnel network. This mapping is essential for the conservation of the paint layer. If the paint layer needs to be consolidated, the restorers need to be certain that the underlying board is solid, so that the paint layer does not collapse (as already happened in the restorations in the early 1900s).

#### From the surface to the inner layers

Generating a global view of the painting that represents the state of the support and its surface required a consistent representation of both the inner structures (tunnels and anomalies of the wooden support) and the external surface (with paint blistering and irregularities). These two datasets were then combined into a single, surface-based 3D representation, which was more compact and easier to observe and measure directly.

While the tomographic data contain a dense representation of the whole painting, working on a large voxel volume is cumbersome. As the needed map only concerns specific elements of this volume, the natural choice was to create a more focused and compact representation of the critical parts of the support. The inner structures of the tunnels and other anomalies were extracted from the tomography volume using a density-based segmentation, refined with diffusion algorithms and manual tweaking. This procedure was done twice, first to extract the *empty* tunnels and low-density detachment areas of the support, and then the *filled* tunnels blocked by dust and wood residues and areas filled with high-density compounds from previous restorations. The two extracted volumes were then converted into surface 3D models, which were then combined with the external surface from the 3D scanning.

**Figure 3:**
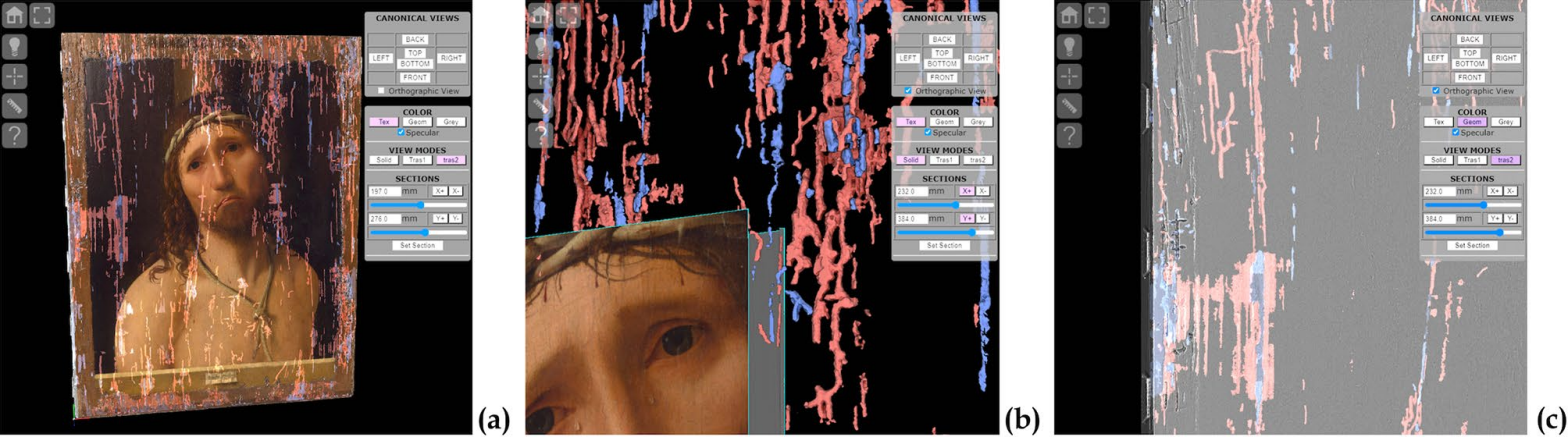
The combined inside and out dataset. (**a**) All the tunnels and anomalies, shown in transparency below the surface. *Empty* and *filled* tunnels are shown in red and blue, respectively. (**b**) The networks of different sized tunnels. (**c**) Loss of cohesion between the wood fibres on the left of the painting.

The combined datasets represent a three-dimensional global map of the painting, thus providing an accurate and effective representation of the inner and outer anomalies, and their spatial relationship. Figure 3 shows the network of *empty* and *filled* tunnels underneath the painting surface (in red and blue, respectively), the differences in the size of the tunnels, the presence of areas where the wood fibres have lost their cohesion and become partially detached from each other.

**Figure 4:**
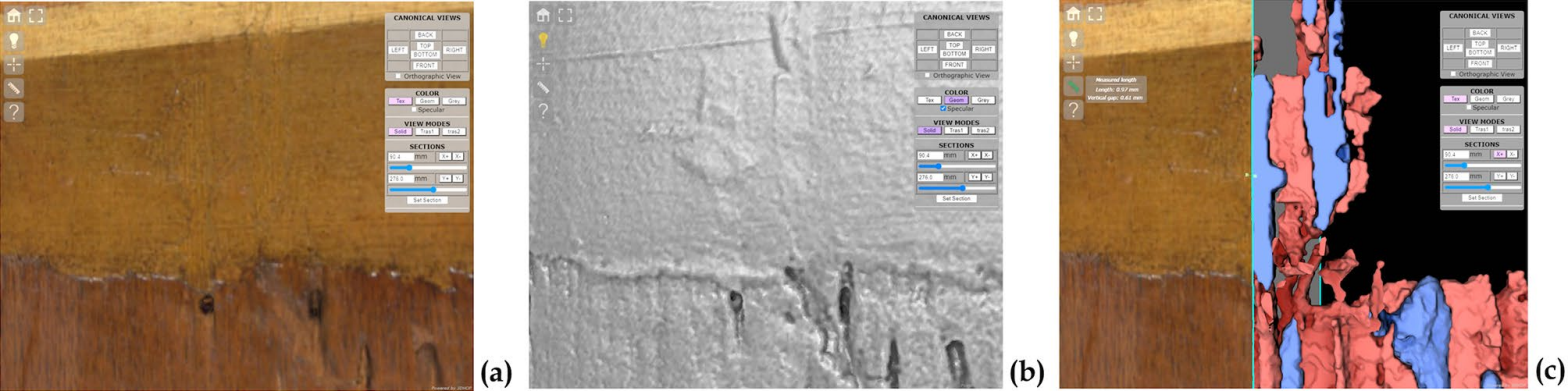
Exploring a previously restored area. (**a**) On the texture, the retouched areas are covered with typical hatching used by restorers. (**b**) A small bump is visible with the detail-enhancing illumination and simulated grazing light. (**c**) Just 0.6 mm below the surface, the collapsed holes that were filled during the past restoration are shown in blue.

The traces left by previous restorations can be inspected and measured thanks to the combination of the colour texture, surface geometry and inner structures. An example is presented in Fig. 4: the colour texture shows the standard vertical hatching used by restorers to fill in an empty area of the painting; a small bump is visible on the surface geometry, especially when using grazing light; the inner structures show, just 0.6 mm underneath the surface, the geometry of the collapsed hole that was filled during the previous restoration, and the still unfilled tunnels nearby.

The technical documentation for this kind of analysis usually consists of colour-coded 2D maps (showing, for each point on the painting surface, numerical values such as the depth of an underlying tunnel, or the level of surface roughness). This, however, is a *static* representation, which only contains a single, predetermined measurement. In contrast, this project used combined datasets to build an *interactive* representation (Figs. 3 and 4), thus making it possible for the restorers to access, inspect and measure every aspect of the geometries, thus fully exploiting their 3D nature.

### The secrets of the workshop: colours and techniques

The investigation of the painting using multiple non-invasive techniques such as macro-photography, HSI, UV-fluorescence, X-ray tomography, and MA-XRF mapping revealed information about Antonello da Messina’s painting technique. The combination of results provides a virtual tour of the painting and its stratigraphic layering, from the choices of the materials for the preparatory layers to the fine technique used to create the bright and extremely naturalistic details.

#### From the preparatory layers to the imprimitura

As highlighted by the X-ray tomography, the pictorial film of the painting is extremely thin: the thickness of the sum of the preparation layers and the pigment layers is about 0.2–0.4 mm. The 3D model highlights two thicker areas, in relation to the crown of thorns and the rope around the neck, most probably related to thicker pigment layers in those areas.

**Figure 5:**
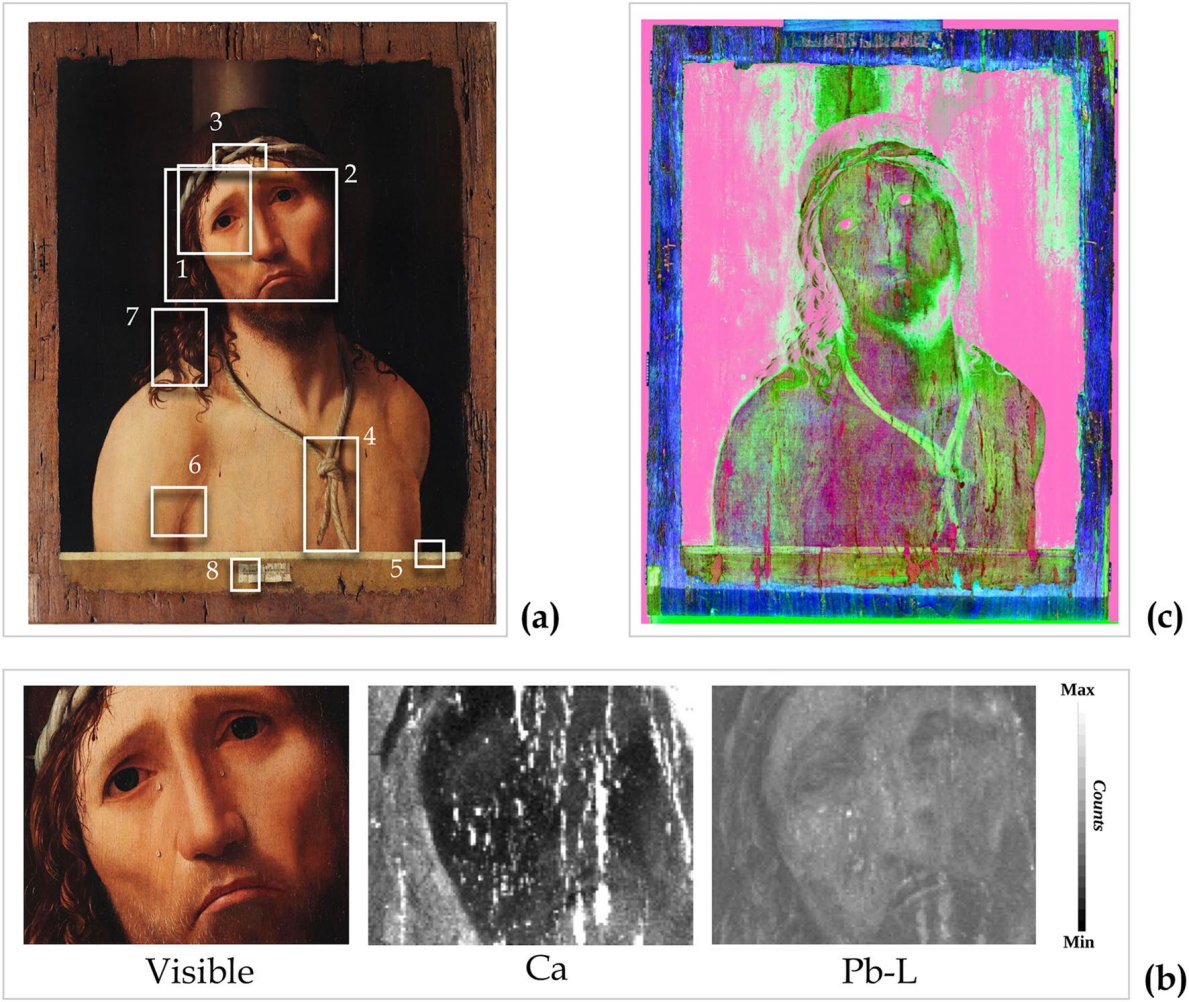
Studies of the underneath layers. (**a**) Visible picture of the painting and the eight areas analysed by X-ray fluorescence (MA-XRF). (**b**) Visible detail and MA-XRF elemental distribution maps of Ca and Pb-L of the *visage* area (13 × 10.3 cm). These results suggest the presence of preparation layers, composed of a Ca-based material and lead white. Note that the Ca distribution also highlights retouches and/or pictorial detachments. **(c)** Hyperspectral imaging (HSI) analysis. False-colour image obtained from the derivative cube of the short-wave infrared range (SWIR) HSI data (R = 1386 nm; G = 1479 nm; B = 1520 nm). The greenish areas map the gypsum and lead white distribution in the underneath layers.

The MA-XRF mapping, performed on eight different areas (Fig. 5a), revealed the widespread presence of Ca and Pb, which are usually related to the use of a Ca-based material and lead white, respectively. For example, the analysis of the *visage* area is shown in Fig. 5b: the Ca distribution shows a low signal in the layers characterized by a high content of Pb and vice versa, producing a "negative image". Usually, this behaviour suggests the presence of a preparation layer, composed of a Ca-based material (likely calcium carbonate or gypsum^7^), overlaid by a Pb-based pigment (likely lead white^8^).

In order to confirm the presence of gypsum and lead white in the preparatory layers underneath the painted surface, the HSI data in the short-wave infrared range (SWIR) were analysed. In fact, both gypsum and lead white feature very typical absorption bands in the 1400–1500 nm spectral range, which, in principle, allow their detection by analysing the spectral reflectance. When these materials are found together this leads to a typical reflectance shape, in which the triplet of gypsum—structured in three sub-bands at 1445 nm, 1490 nm, 1540 nm—is modified by a strong and widened band at 1450 nm due to lead-white^9^. However, as with other paintings by Antonello da Messina, such as the “Trivulzio portrait”^10^, in Ecce Homo a very dark background surrounds the main figure of Christ. This dark paint film strongly absorbs the incident light, thus resulting in a very low reflectance signal, which is not suitable for identification purposes. Nevertheless, the variability in the pictorial thickness enabled the underneath layers to be detected, where the surface pictorial film is thinner or more degraded (Supplementary Figure S1 online).

By calculating the derivative cube in the SWIR range, and selecting suitable bands (R = 1386 nm; G = 1479 nm; B = 1520 nm) in correspondence of inflexion points of the typical gypsum and lead white spectral shape, a false colour image was obtained, as shown in Fig. 5c. In this map, the greenish areas correspond to the spectral markers of gypsum and lead white, which belong to the inner layers and therefore are only detectable where the dark paint film is weaker. The sharp green contour around the right shoulder of the Christ also confirms that the dark pigment of the background lies above the preparation layers. As expected, markers of lead white were associated with other areas, such as the rope. This image is fully consistent with the MA-XRF findings on the Pb element, associated with the use of lead-white as both pictorial material and preparatory layer.

Therefore, the combination of MA-XRF and HSI results indicated a preparation technique that was fully consistent with the traditional practice of Renaissance panel paintings^11,12^: a white or toned *imprimitura* (here lead white) was applied to create a ground over a primer (here gypsum—CaSO_4_ 2(H_2_O)), in order to reflect the light through the painting layers^8^, almost creating a 3D effect. These results are in agreement with other studies on this Ecce Homo painting^5^ and other artworks by Antonello da Messina^13^.

#### From a draft to a finely detailed figure

**Figure 6:**
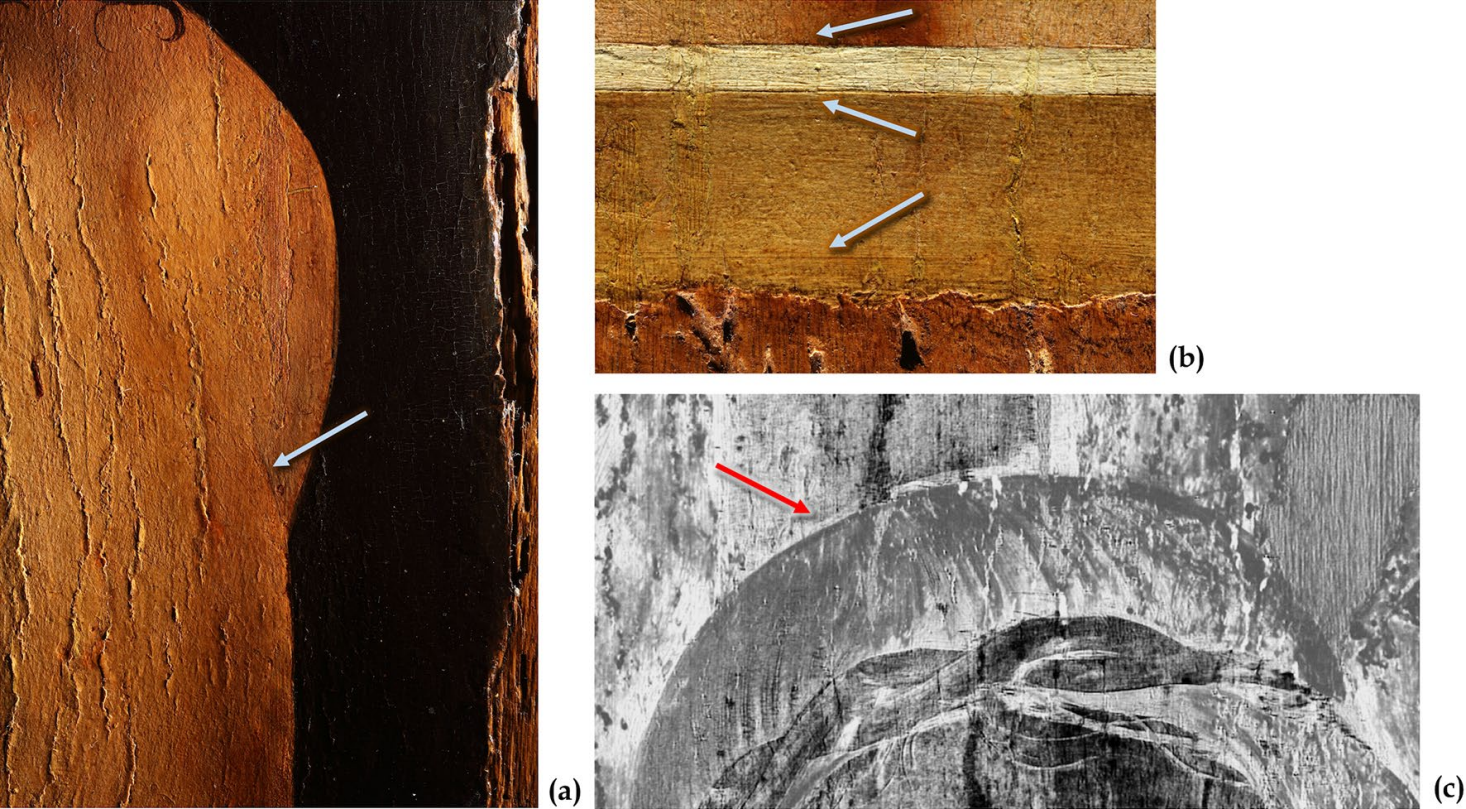
The use of faint incision as a reference for the torso and the parapet and imaging of a *pentimento*. (**a**) Details in visible light of the slight incisions made to delineate the torso, (**b**) the parapet and as a reference for positioning the frame (photos by Carlo Pagani). (**c**) Detail of the upper part of the painting in the infrared region where a *pentimento* is visible, with a change in the position of Christ’s head.

The hyperspectral imaging analysis in the infrared region did not reveal any preparatory drawing, although the macro-photography in visible light highlighted incisions used to delineate a few essential traits. In particular, these faint incisions are visible to define the torso of Christ, to delineate the parapet, and as a reference for the frame positioning (Fig. 6a,b). These results confirm the spared use of drawing by Antonello da Messina, in agreement with other studies on his painting technique^5,13,14^.

Despite the absence of a preparatory drawing, the imaging in the infrared region revealed a *pentimento*: in the upper part of the painting a change was visible in the position of Christ’s head, which originally has been placed further up (Fig. 6c).

Overall, the multimodal investigations highlighted that Antonello da Messina’s technique is characterized by the creation of the highly detailed figure of Christ, starting from an early outlined figure, and adding subsequent highlights, focused on the foreground and on the most illuminated areas.

The tomographic slices related to the pictorial layers, as shown in Figs. 1c and 2a, give the first strong indication about it.

The investigation shows a thin and uniform layer, compatible with the hypothesis of an inner layer containing also Pb. Moreover, it reveals a high contrast between the less absorbing dark background—probably due to upper pigment layers characterized by low-atomic number elements—and the more absorbing and clearly discernible figure of Christ, as expected from the use of pigments characterized by high-atomic number elements such as lead white. The use of a thin lead-white inner layer, covered by a pigmented dark layer to make the background and exploited as a light base to depict the figure, is in line with other studies on Antonello da Messina’s paintings^13^.

**Figure 7:**
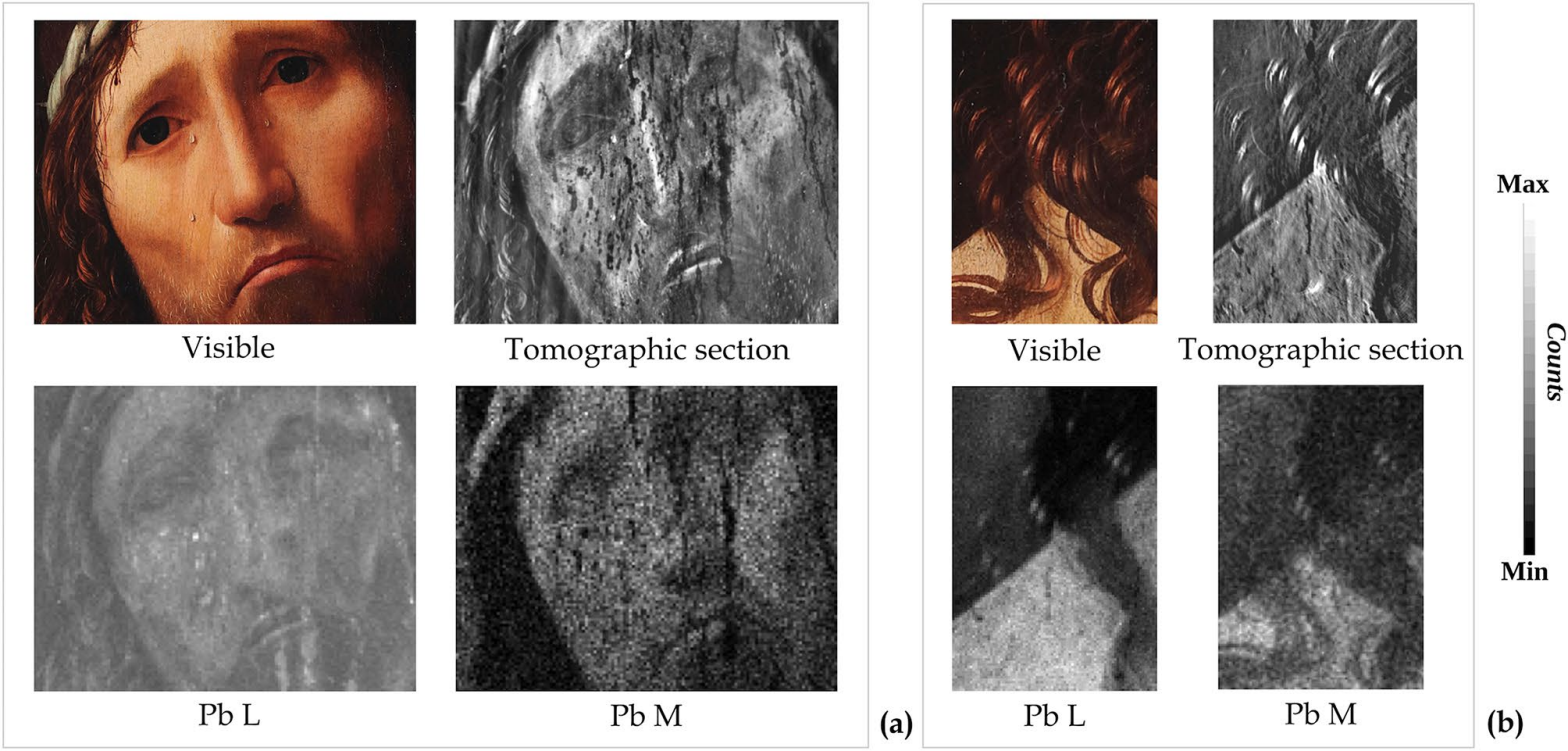
Analysis of the *visage* and right shoulder. Comparison of the visible image, tomographic section and X-ray fluorescence (MA-XRF) elemental distribution maps (Pb-L and Pb-M) of (**a**) the *visage* area (13 × 10.3 cm) and (**b**) the right shoulder detail (4 × 6 cm).

This interpretation is further strengthened by the detailed analyses of the MA-XRF distributions of the signal intensity for different Pb energy line emissions (Pb-L and Pb-M). As shown in Fig. 7a, the MA-XRF mapping of the *visage* (area 2 of Fig. 5a) shows the widespread use of lead white in the subsequent layers of the painting. In fact, the difference between the distribution of the low energy Pb-M, from the shallowest painting layers, and that of the high energy Pb-L, which combines the contribution of inner and shallower layers, reveals two different applications of lead white.

Stronger evidence of Antonello da Messina's painting technique is given by the examination of the right-shoulder detail of the Christ figure (area 7 in Fig. 5a), as shown in Fig. 7b. The tomographic section, and more clearly the Pb-L map in Fig. 7b, show that the draft of the torso had been gradually refined with lead white, leaving an empty zone for the curls and, subsequently, the hair was painted using low-absorbing pigments and highlighted with high-absorbing pigments.

In addition, an asymmetry is evident between the right-hand side of Christ’s *visage*, which is extremely detailed and perfectly readable in tomography, and the left side, which has only been sketched. This effect is most probably the artist’s stylistic choice and part of his painting technique: the figure is lit by a sidelight from the right, which gives shapes to both the face and body.

These results provide further confirmation of Antonello da Messina's painting technique, with the key role played by the light and its effect^10,13^.

#### The colour choices

Combining the MA-XRF mapping results with the HSI imaging made it possible to identify the pigments used for the Ecce Homo painting, highlighting Antonello da Messina’s narrow pigment palette and his skilful use of a mixture of pigments to obtain the characteristic rich shades.

The HSI analysis on the entire surface provided evidence of the use of a precise selection of pigments to reproduce the flesh tones. The reflectance spectra extracted from different areas showed that the flesh tones were depicted using mixtures of white lead with iron-oxide based pigments (likely ochres and earths), as well as with vermilion, with a keen choice and variation in the concentrations of the different pigments to attain the chiaroscuro and sculptural effect.

**Figure 8:**
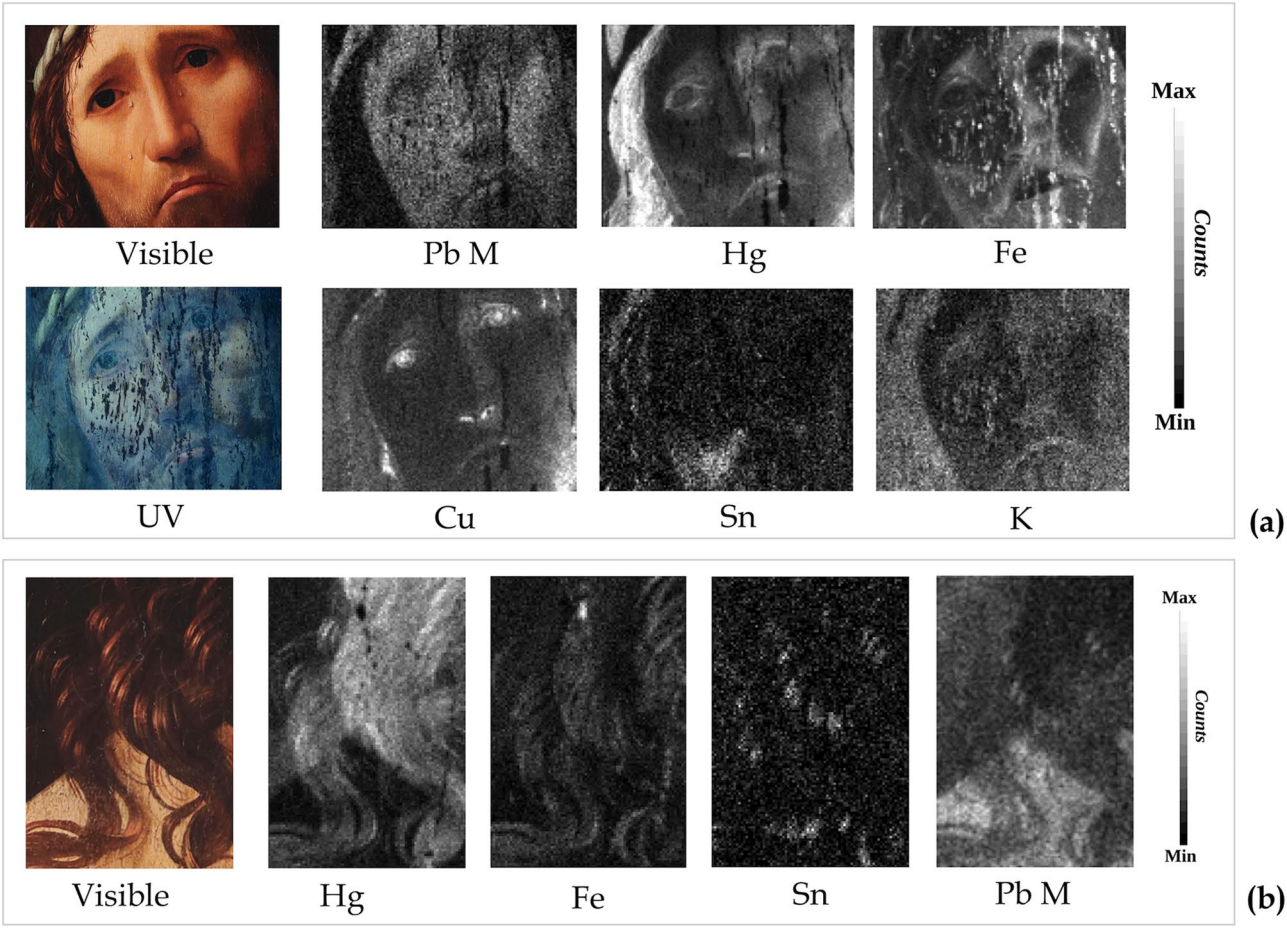
(**a**) Analysis of the Ecce Homo *visage* (13 × 10.3 cm). Picture in visible light and ultraviolet fluorescence (UV) image of Christ’s *visage* and elemental maps of the same area obtained by X-ray fluorescence (MA-XRF) mapping analyses. (**b**) Analysis of the right shoulder (4 × 6 cm). Picture in visible light and elemental maps of the same area obtained by X-ray fluorescence (MA-XRF) mapping analyses.

The MA-XRF scanning analysis of the Christ *visage* (area 2 in Fig. 5a) in fact revealed the presence of Pb, Hg, and Fe (Fig. 8a, and Supplementary Figure S2 online), thus confirming the use of different mixtures of lead white with vermilion and traces of iron-oxide based pigments.

In the shadow areas, coloured from red to brown, the MA-XRF analysis revealed higher signals of Hg (Fig. 8a-Hg) and Fe (Fig. 8a-Fe). The analysis also detected Cu (Fig. 8a-Cu) in the darkest black areas and in the finest details, such as the irises, nose, nostrils, and mouth. These results suggest the use of vermilion, earths, ochres, and a copper-based material, probably mixed in different proportions or applied in sequential steps to obtain different hues, from mid to very dark tones, which were used to define the facial features.

A similar use of the shades, made from vermilion and iron-based pigments, was found in the hair curls, (Fig. 8b-Hg,b-Fe). These results were all confirmed by HSI spectral analysis.

In addition, a comparison of the MA-XRF Sn and Pb-M maps clearly highlights the simultaneous presence of these two elements in the lighter curls (Fig. 8b), suggesting the use of lead–tin yellow to obtain the light blond colour. Sn was also detected by MA-XRF in some details of the beard (Supplementary Figure S3 online) and crown, suggesting the use of the same pigment.

The other highlights, as mentioned in the previous section, were mainly created with successive small lead white strokes. The MA-XRF analyses and the HSI reflectance data show that lead white was used to depict some whitish elements (crown, rope, and cartouche).

The distribution of potassium, also reported in Fig. 8a-K, becomes clearer when compared with the UV fluorescence imaging of the same area (Fig. 8a-UV). While the broad distribution of K seems related to the varnish, which is responsible for the diffused fluorescence observed in the UV image, the K spots detected in the right cheek area highlight an evident correlation of this element with some of the retouches.

The brighter spots in the Fe map, present on Christ’s *visage* and matching the low-fluorescence darkest areas of the UV image (Fig. 8a-Fe and 8a-UV), can be identified as retouches. The use of different pigments for similar retouches (as found on the K and Fe maps), indicates that there was more than one restoration phase, or different restorers and epochs, in line with findings from other studies^5^.

**Figure 9:**
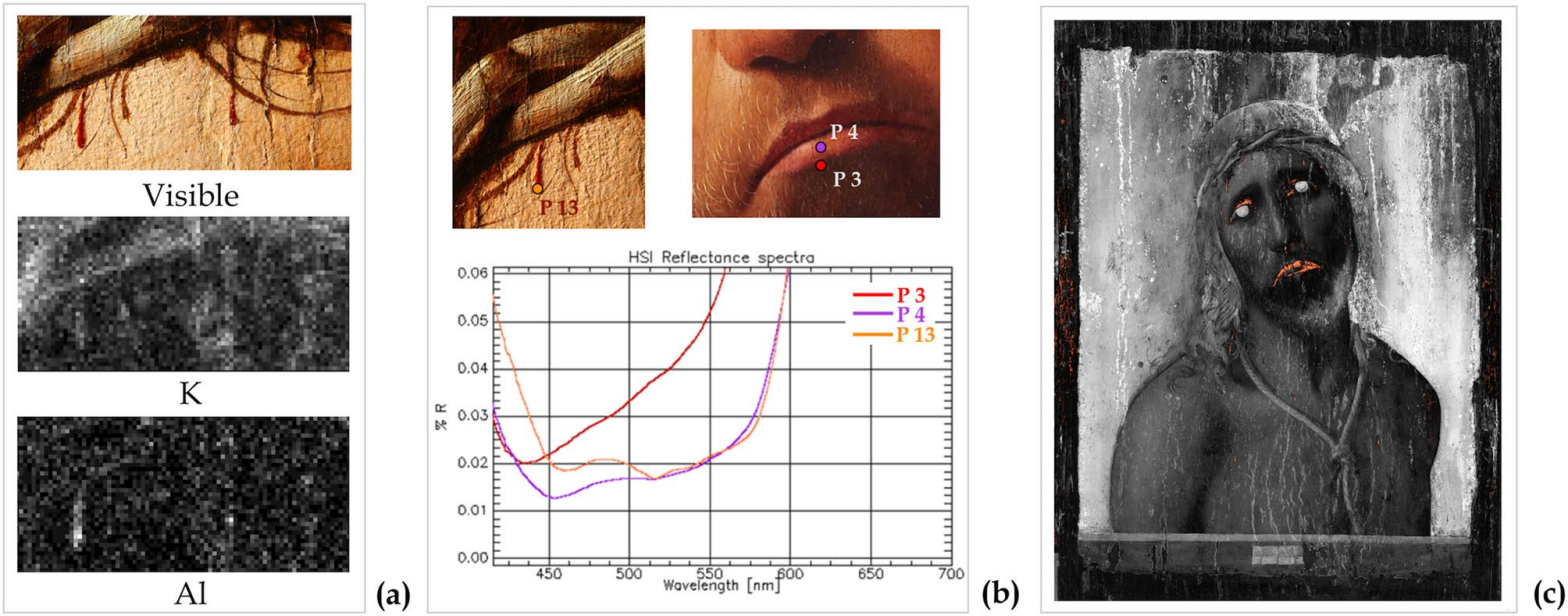
Investigations of the blood drops and lips. (**a**) Visible detail (photo by Carlo Pagani) and X-ray fluorescence (MA-XRF) elemental distribution maps of K and Al in the blood drops (4 × 1,8 cm). (**b**) Visible details and reflectance spectra extracted from the hyperspectral imaging (HSI) data cubes on the drops (point 13) and on the lip (P3 and P4). **(c)** Classification map overlapped with the spectral angle mapper (SAM) rule image where the orange-coloured pixels are classified as being similar to the spectrum on the lip (P13), identified as red-lake.

The analyses of details such as the blood drops and the lips revealed interesting features. The MA-XRF mapping analysis of the blood drops on Christ’s forehead, shown in Fig. 9a, was acquired by enhancing the detection of light elements with a He flow. Both potassium and aluminium were found in some of the blood drops, suggesting the use of a red lake such as potash alum (K-Al sulphate)^15^. The analysis of the HSI reflectance spectra extracted in correspondence of the drops (P13), confirmed this hypothesis (Fig. 9b). This spectrum clearly shows the typical spectral features of the red lake in the 500-550 nm range, with a strong absorption band structured in two sub-bands at about 515 nm and 530nm^16^. However, although these diagnostics bands were clearly detectable in the blood drop spectrum (point P13), they appear very weak (point P4), or almost absent in other similar red shades, such as the lower lip detail (point P3), which instead prevalently shows the typical S-shaped behaviour of vermilion (Supplementary Figure S4). In order to better understand the use of the red-lake and to map its overall distribution and occurrence, the spectral angle mapper (SAM) classification was performed on HSI data, using the P13 spectrum as reference end-member, and setting the angle threshold (maximum angle) at MA = 0.09 radians. The corresponding classification map, shown in Fig. 9c, clearly shows the meticulous use of red lake to finish only selected details, such as the blood drops and the eye contours, or to veil the vermillion red of lips. This SAM distribution map clearly shows that, unlike the other red-pigments, red-lake was exclusively used for glazing or painting fine details (drops), thus confirming Antonello da Messina’s skilful and systematic use of the glazing technique^5,15^.

The SAM analysis of red-lake revealed further insight into the painting technique. In the grey-scale rule image, reported in Fig. 9c, darker pixels correspond to smaller spectral angles with the reference (the P13 spectrum, identified as red-lake), thus locating areas spectrally similar to this material^17^. Thus, the zones represented as darker in the background, which also correspond to a thinner paint layer (see also Fig. 5 and related discussion), indicate the presence of a reddish layer, likely made of red lake laid down—or mixed with—the *imprimitura* (in agreement with other studies on Ecce Homo painting^5^). This practice was common in Renaissance painting technique^18^.

Although MA-XRF and HSI spectroscopy are not the most appropriate techniques to identify black pigments, even the very dark areas that looked black, were investigated, such as the signature on the cartouche, pupils and background.

**Figure 10:**
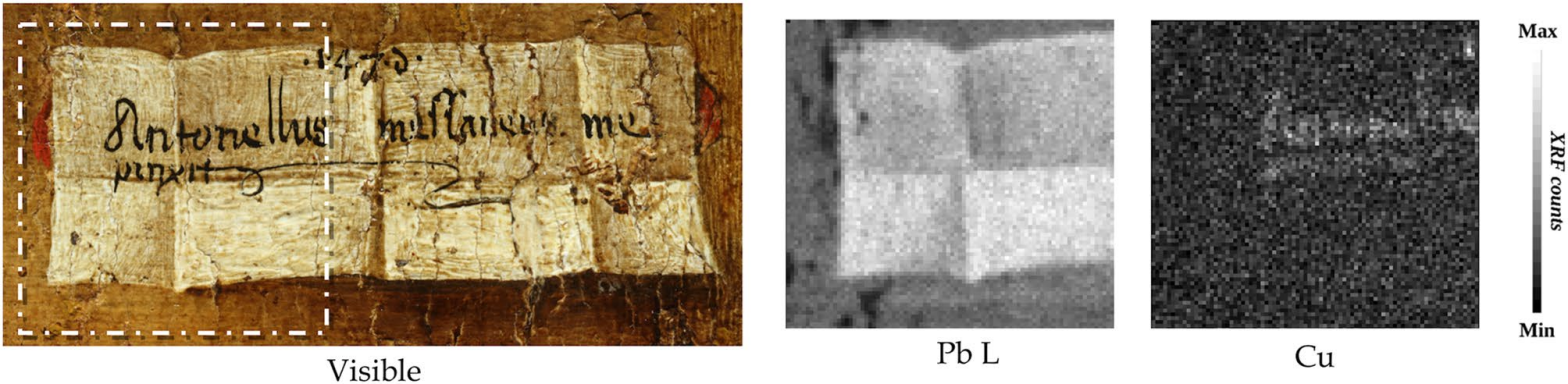
Analysis of the cartouche. Picture in visible light (photo by Carlo Pagani) of the cartouche and the distribution of Pb L and Cu obtained by X-ray fluorescence (MA-XRF) mapping analyses, related to the sub-area highlighted with dots (2.5 × 2.1 cm).

Figure 10 reports the detail of the crumpled cartouche. The black writing on a white background is the author’s signature in Latin: «*Antonellus messaneus me/ pinxit*», followed by the year. Unfortunately, the last number is illegible, making dating difficult.

In this area, the lead white used to paint the cartouche absorbs the contribution of the X-rays from the inner layers (Fig. 10-Pb *L*), avoiding interference from the characteristic signals of the materials of the underneath layers and thus enabling an easier characterization by MA-XRF of the black material on top. Only the Cu map (Fig. 10-Cu) shows a clear correlation with the black signature, indicating the presence of a copper-based material in the black colour. However, since XRF analysis is unable to detect organic black pigments, a possible mixture or overlap with an organic black component, which cannot be revealed by XRF, cannot be excluded.

In order to gain further insights into the dark pigments on the painted surface, HSI data were analysed. As expected, the reflectance signal of all the dark areas (e.g., background, and pupils) was very low and noisy, thus preventing a reliable identification of the pigments used. However, after pre-treatment of data aimed at smoothing and denoising the signal, a very weak reflectance peak around 500 nm could be singled out, which is in line with the hypothesis of the presence of a Cu-based material.

The combination of results obtained with MA-XRF and HSI highlight that a Cu-based material is a ubiquitous component of the black areas analysed. Although they do not provide an exhaustive identification of the dark pigment, these findings are fully consistent with the analyses of other paintings by Antonello da Messina^13,15,19^.

### What remains: imprints on the painting

The elaboration of HSI data brought to light further interesting and peculiar details.

**Figure 11:**
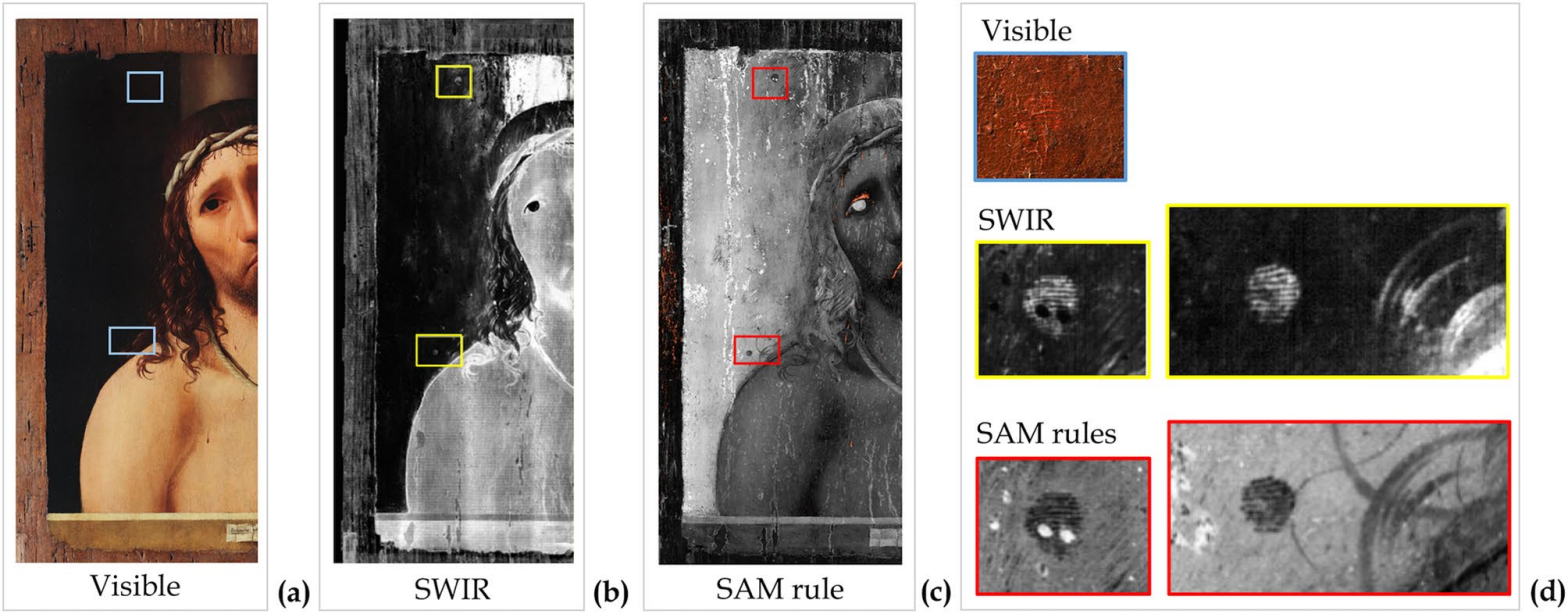
Fingerprints. (**a**) Location of the traces, not visible in the painting. (**b**) Evidence of fingerprints as lighter traces in the image extracted from the short-wave infrared range (SWIR) derivative cube at 1480 nm. (**c**) Evidence of fingerprints as darker traces in the grey-scale spectral angle mapper (SAM) rule of red-lake. (**d**) Magnification of: upper fingerprint in visible macro-photography (photo by Carlo Pagani); upper and lower fingerprints in SWIR and SAM rule.

The analysis of the near-infrared region (900–1700 nm) revealed two traces that were almost invisible to the naked eye and closely resembling human fingerprints. As shown in Fig. 11, these traces are located on the right of the Christ figure and were clearly visible in both the image extracted from the SWIR derivative cube and in the SAM rule elaborated image**.** These traces were further investigated with macro-photography confirming two human fingerprints on the dark background layer, but underneath the varnish layer. The position of the fingerprints in the painting stratigraphy would seem to indicate that they were left shortly after the laying of the dark pigment in the background when it was not yet completely dry.

In addition, in Fig. 11, traces of the papillary crests appear orange in the macro-photography (visible), thus revealing a reddish layer -underneath the dark background- over or mixed with the lead white-based *imprimitura*. This confirms the interpretation of the SAM rule image reported in the previous section.

On the head, macrophotography revealed a series of very small arrowhead signs, probably left by the sleeve of some clothing that touched the still fresh colour. Here, too, the imprints appeared orange-coloured.

### Beyond the analyses: disseminating science

The analytical data gathered during scientific investigations have been also exploited for exploring the data publishing scenario. In this context, the multimodal datasets were used as an informative layer to build two applications: a web platform, intended for technical users and aimed at enabling specialized access to project data, and a museum kiosk, addressed for the general public and aimed at opening the project results to a wider audience.

#### Sharing data, from expert to expert

The raw data collected in the project are a resource that can be used to provide restorers and experts with new perspectives regarding particular features of the painting. For this purpose, a technical/specialized data publishing pattern was explored. In this framework, it was chosen to use the 3D datasets related to the painting support (both the surface digitization and an X-ray tomographic analysis) in order to develop a tool giving access to the global map resulting from their integration. Created as a web-based interactive platform [http://vcg.isti.cnr.it/activities/eccehomo/insideout], this demonstrator is able to display the geometry of the painting, and also to enable it to be studied and measured in real-time (Figs. 3 and 4).

The “inside-out” viewer uses the 3DHOP tool^20^: developed by the ISTI-CNR research group, it is an open-source framework for the creation of interactive web presentations of high-resolution 3D models. Thanks to its configurability, it was possible to create a highly specialized viewer. Its multiresolution engine enables the use of a textured 45 million triangles model for the painting, and a further 7 million triangles for the tunnels, while still ensuring a fast network transfer and optimal rendering speed.

The platform has multiple visualization modes, and each component of the visualization has multiple parameters. With this "sandbox" approach, the user is free to experiment with the visualization modes and parameters, to find the perfect combination of parameters in order to see, measure, and document the areas of interest.

**Figure 12:**
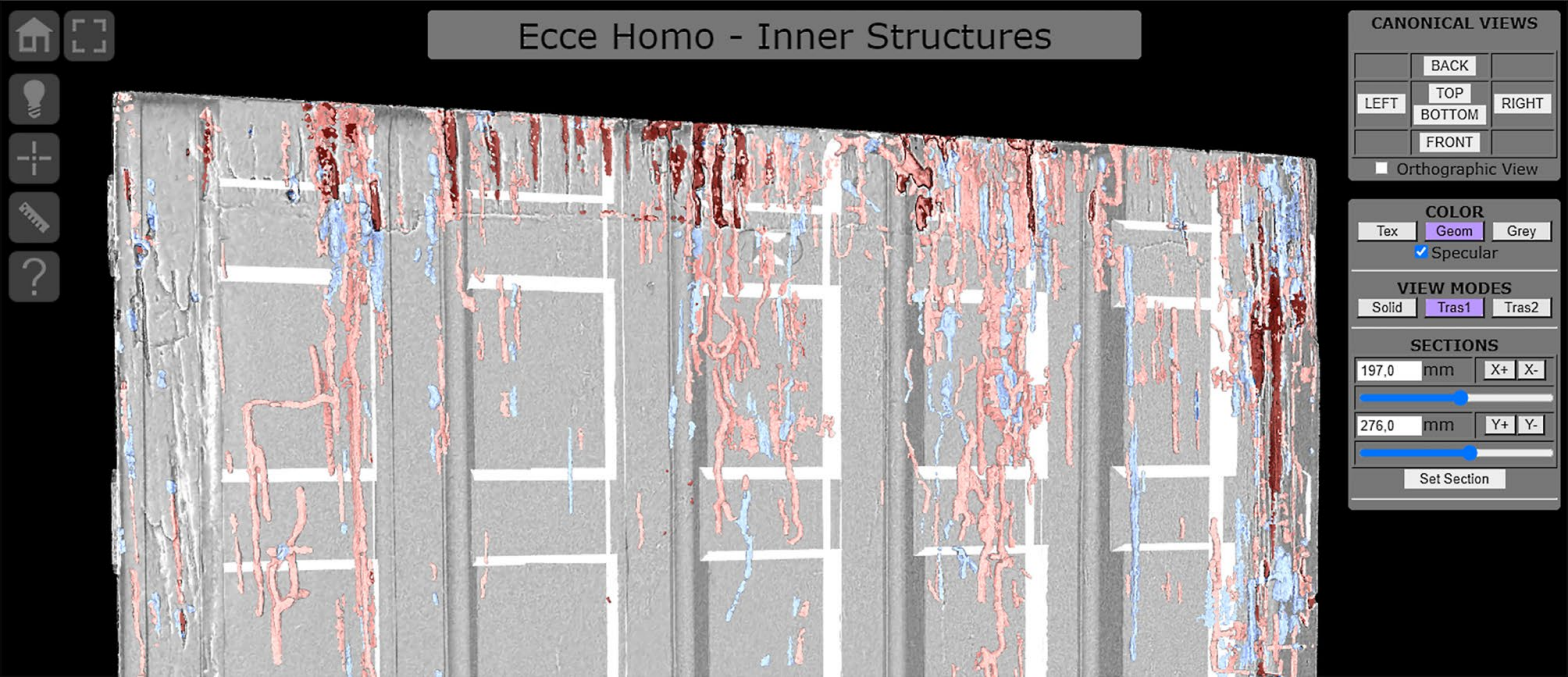
The geometry of the painting and its internal structures, visualized using one of the rendering modes provided by the technical platform. This figure shows the details of the interface designed for expert users. Top-left: a toolbar with basic interaction/navigation features, two measurement tools, and a button to control the lighting. Top-right: a panel with the camera setup (predefined canonical views and orthographic projection), rendering fine-tuning (transparency and colour modes), and cut-through sections control.

The user can, interactively, control the point of view and rendering style of the 3D objects (Fig. 12). Anomalies on the outer surface are made more perceivable using different colour mappings and rendering techniques, and the controllable lighting can be used to simulate "grazing light" inspection. To expose the inner structures and perceive their relationship with the outside surface, different transparency effects can be used, in combination with sliding cut-through sections. The user can also take point-to-point and depth measurements across the whole 3D dataset.

The utility of this web platform is twofold: it enables the scientific community not directly involved in the project to access the collected data, but it was also a clever way of sharing the data among the various partners of the diagnostic project, creating a shared workspace between physically distant workgroups.

#### Spreading knowledge, from expert to the general public

Research data can also play an important role in sharing knowledge or in creating public interest. For this purpose, a non-expert/generalist data publishing pattern was explored, too. Aimed at a museum audience, this activity resulted in a multimedia platform designed for disseminating project results to a wider public.

**Figure 13:**
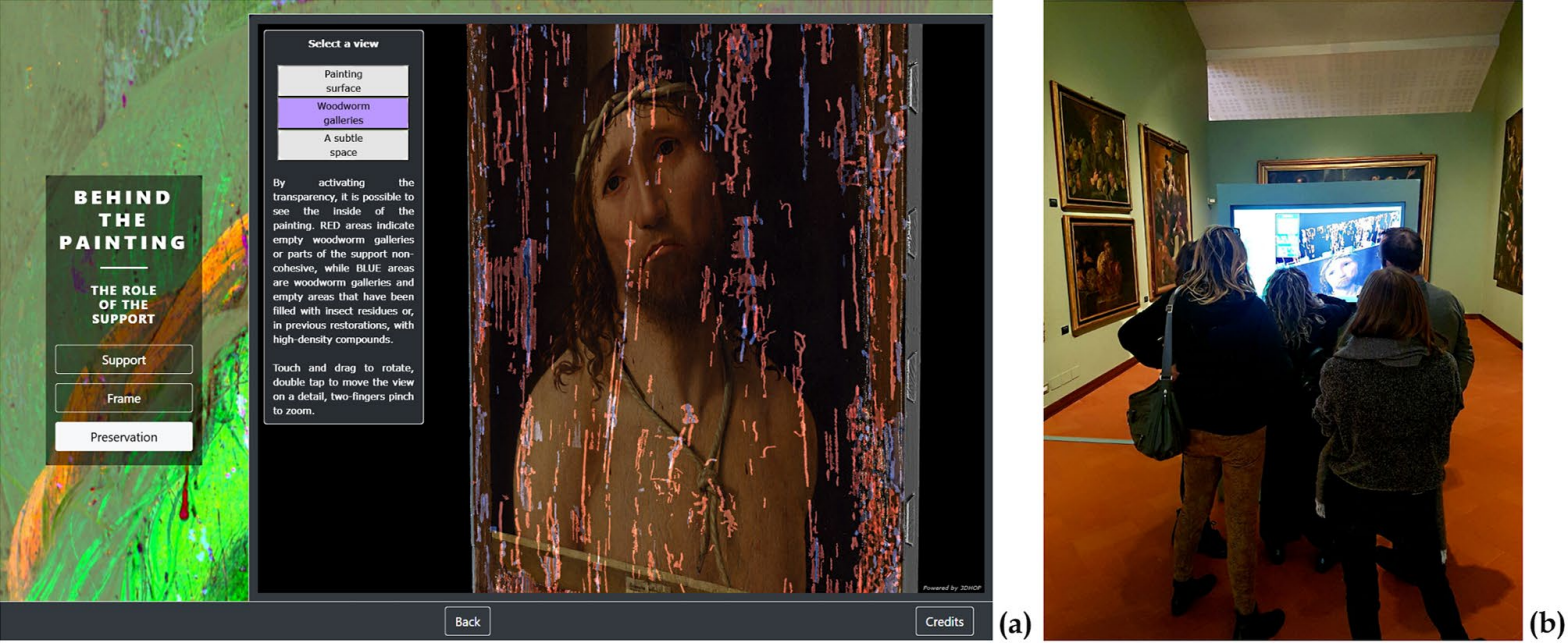
The multimedia kiosk on its inauguration (November 2019). (**a**) A kiosk panel showing the simplified interface of the 3D viewer. Instead of metric tools, three buttons correspond to three bookmarked views that guide visitors through the tunnels and the details of the painting support. (**b**) Visitors interacting with the 3D model through the kiosk touch screen.

**Figure 14:**
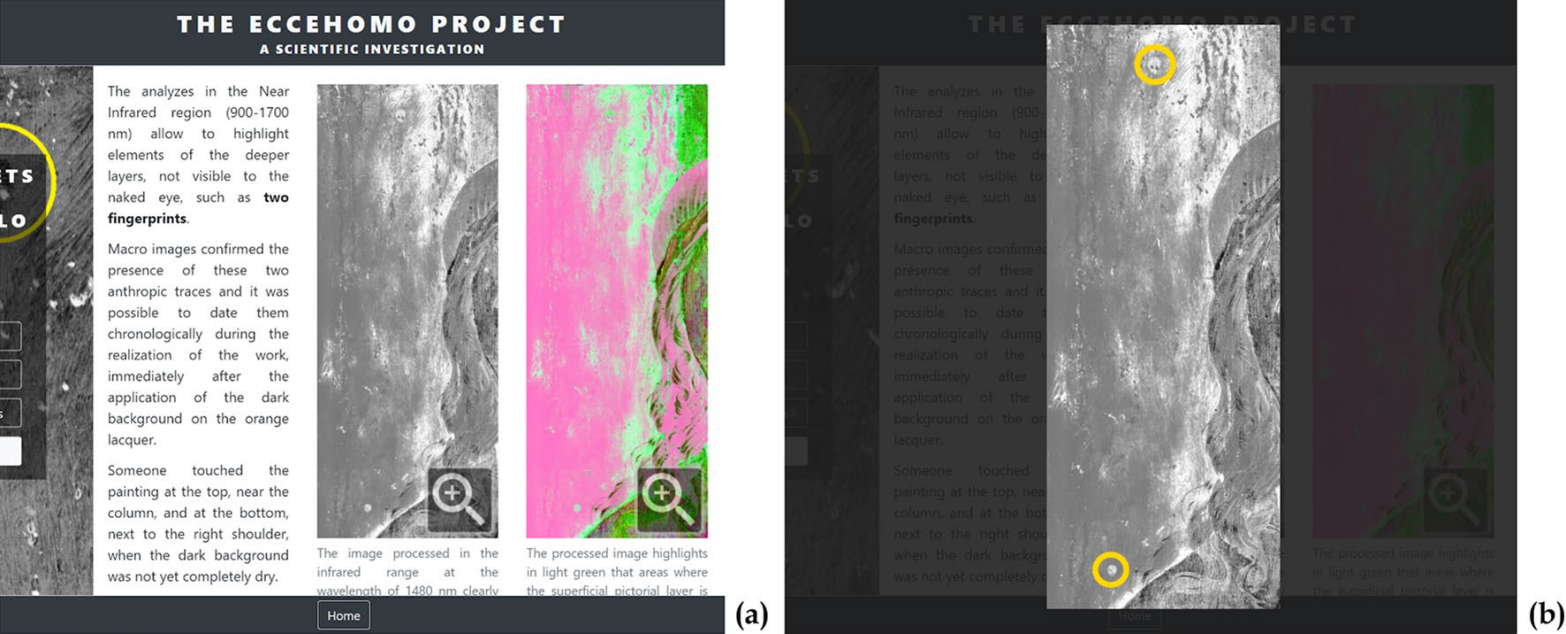
Kiosk panel showing text and images mixed together for presenting to a general public the scientific analysis on the pictorial layers. This figure shows an example of an interactive 2D image enriched with visual elements: by clicking on the image extracted from the short-wave infrared range (SWIR) derivative cube at 1480 nm (**a**, grey-level figure), the image is magnified showing the location of the fingerprints (**b**).

Despite being more conventional, this platform works very well in showing a further, and easy to achieve, exploitation possibility for scientific data. It is based on the analytical datasets collected in both 2D and 3D investigations, adapted to provide accessible content to users with heterogeneous expertise. This content is presented through a storytelling path organized in four thematic sections:

1. “The artwork in digital—Exploring the Antonello’s world”, which contains a 2D and 3D high-resolution digital replica of the painting.
2. “Traveling through time—From the workshop to the museum”, which presents the most important information on the Ecce Homo story, organized in a chronological path starting in the artist’s studio and continuing to the present day.
3. “Behind the painting—The role of the support”, which shows the results of the investigations on the wooden panel, including the high-resolution 3D map of its inner structure, presented with a simplified interface that provides a virtual tour through a selection of predefined views (Fig. 13a).
4. “The secrets of the Master—Colour and materials”, which introduces the most interesting findings of the analysis of the pictorial layers, presented through a selection of 2D images enriched with visual elements and informative texts to guide the users in their interpretation (Fig. 14).

Developed using web technologies/languages, this platform can be accessed both as a museum kiosk (running on a local webserver) and remotely as a web application (running on a standard webserver). To enable the visualization of the high-resolution 2D and 3D assets, it exploits the Relight open-source library^21^ (developed by the ISTI-CNR research group, provides performing multiresolution streaming and rendering schemes for hi-res 2D data) and the already introduced 3DHOP framework.

The multimedia platform was presented and exhibited as a kiosk (Fig. 13b) in a public conference dedicated to the “ECCEHOMO” project, which was very well received by the public.

## Conclusions

The non-invasive and multidisciplinary investigation of Antonello da Messina’s masterpiece “Ecce Homo” provided detailed scientific documentation of the painting. This knowledge, coupled with the wise conservation practices and restorer’s experience, led to an enhanced comprehension of this fragile artwork.

Data-fusion from multiple 2D and 3D analytical techniques not only provided experts with new data but also led to creating a virtual tour of the painting, from the inner to the outer layers, thus offering a novel, and attractive way to disseminate the results to the widest public.

The 3D investigations revealed the exact positioning of the internal and superficial woodworm tunnels and pictorial flaking, thus creating a global view of the painting and highlighting the consequent fragility of the superficial painting layers.

Integrating X-ray tomography, MA-XRF mapping and HSI imaging revealed the painting technique and the artist’s choice of colours.

The investigations highlighted some elements of the creative process of the Ecce Homo, with the main role played by the use of light. The light on the right-hand side was used to model and define the figure of Christ in contrast to the uniform and dark background. This technique is clearly visible in the asymmetry between the right-hand side of Christ’s *visage*, which is extremely detailed, in contrast with the left side which is only sketched. Starting with just an outlined figure, Antonello da Messina created the extremely detailed figure step-by-step, adding subsequent lead white highlights, on the right-hand side, the most illuminated areas, leaving the left-hand side with fewer details.

The richly shaded palette used to create the Christ figure was created with different mixtures or the superimposition of lead white, iron-oxide pigments and vermilion, whereas red-lake was used exclusively to glaze or depict small details (blood drops, eyelids and lip shadows). In the darkest and finest details, such as the pupils and nostrils, as well as in the background, a Cu-based material was used. The masterful use of the shading palette, with the powerful hues from middle- to very dark-tones, creates the exceptional *chiaroscuro* and sculptural effect.

The three-dimensional effect is also enhanced by the painting highlights, mainly created with small successive lead white strokes and, for the hair, beard and crown, with the lead and tin yellow pigment to obtain the light blond nuances.

Overall, the body of knowledge and experimental protocols were combined in two different data publishing actions aimed at fostering access to the project results.

The investigation outcomes were presented in a museum kiosk that was exhibited in a public conference dedicated to the ECCEHOMO project. The kiosk generated great interest from visitors, confirming that these kinds of interactive presentations can work very effectively in the public arena.

A technical web platform was instead designed to enable access to the 3D global map of the painting, providing innovative and specialized tools for investigating this digital replica. The “inside-out” viewer demonstrates that interactive explorations of scientific datasets can be very useful even in the technical scenario.

This last contribution can also be seen as the basis for developing more sophisticated tools, which go beyond pure data visualization. Future studies may explore the possibility to integrate the technical web platform with interactive data enrichment/annotation components, that would transform it into a fully qualified system capable of associating the scientific data with the analytical study process.

Moreover, additional scientific investigations may be conducted to study and map the materials used in several past restorations, thus improving the understanding of the current state of conservation and creating guidelines for future interventions.

## Methods

### Data fusion

Data-sharing and data-fusion are some of the main strengths of this work. The project combines all the analysis results in order to derive more structured insights from the cross-referencing of different types of data.

When cross-referencing the data from multiple sensors, the main problem was that each dataset used a different reference space. An alignment step was thus needed to ensure a common reference space for the whole project. As some analyses were three-dimensional and others two-dimensional, the alignment was carried out independently in the 3D space and in the 2D space, and the common 2D space was then referenced to the common 3D space, to bring all the analyses together.

The 3D analyses were aligned by exploiting the common geometry between the 3D scanning and tomography (i.e., the external surface). The geometric alignment produced a rigid roto-translation superimposing the two datasets; the origin and axes were chosen in order for the frontal plane of the painting to be as straight as possible. For the 2D analyses, the HSI elaborated colour image was used as a common reference space, as it covered the whole painting. XRF raster data were scaled to a common resolution, rotated and translated to match the hyperspectral. The 2D data were then referenced on the 3D geometry using an orthographic projection, which was also used to create the texture mapping.

### 3D digitization

Structured-light is a 3D scanning technology suited to paintings, as it has sufficient precision and resolution to effectively measure the minute details of the surface geometry and, compared to lasers, works better on dark and glossy paints and varnishes which notoriously reduce the quality of the measured data^22^.

A GOM Atos 5 M scanner was used, which is a top-of-the-line metrology measurement device. These instruments, designed for precise industrial measurements, are also frequently used in the cultural heritage field. The GOM scanner uses a blue LED light, and can be configured to cover larger or smaller work areas at different resolutions. In this case, it was set to work with a field of view of 30 × 20 cm, with a sampling rate of 0.12 mm. Structured-light devices do not require specific environmental conditions; however they may suffer from strong ambient illumination: the 3D digitization was thus carried out in the room where the painting is normally kept, with most of the lights turned off.

The 3D digitization covered the whole painting: the intricate wooden support on the back of the painting required more scans than the front, which artistically is more important but geometrically less complex. The scanning took around 3 h, generating around 100 range scans. The range scans were initially aligned in the GOM software during acquisition. To help this process, markers were placed next to the object: their location was automatically detected, resulting in a more rigid and deformation-free alignment. This initial alignment was then refined using the ICP algorithm in MeshLab^23^.

The 3D data gathered were cleaned using the GOM software and then MeshLab, removing the unwanted areas (i.e., table, and easel) and parts of the scans affected by specular reflections and noise due to the difficult optical behaviour of the painted surface. The cleaned scans were used, in MeshLab, to create a triangulated surface for the whole painting. The Screened Poisson merging algorithm^24^ was used to obtain a detailed yet noise-free surface. The full-resolution model is 180 million triangles, at a final resolution of 0.1 mm.

Unfortunately, most metrology scanners are unable to capture colour information, and the GOM Atos is no exception. For this reason, an RGB calibrated image reconstructed from HSI Vis data (provided by CNR-IFAC), was mapped onto a 45 million triangles geometry, creating a texture-mapped 3D model.

### X-ray computed tomography

X-ray computed tomography (CT) is a powerful non-invasive imaging technique that gives information on the inner morphology of cultural heritage artefacts and on the manufacture, function, and conservation status of the object^25–28^. With CT a complete 3D reconstruction of the object analysed is possible, thus overcoming an important limitation of radiography, i.e. the superimposition in the image of elements belonging to different layers of the object. CT is versatile, and thus can be applied with good results to different materials and items, such as paintings, ceramics, wooden artefacts, musical instruments and manuscripts^29–34^. Traditionally restricted to medical structures or specialized laboratories, today CT is available in situ thanks to the development of portable and flexible instruments^35,36^.

The analysis was performed with a portable instrument designed for in situ artwork investigations at the X-ray Imaging Laboratory of INFN-CHNet (Bologna). The main components of the CT system are independent, making it suitable for small samples and even entire paintings.

A microfocus 130 kVp X-ray tube by Kevex (mod. PXS10) and a flat-panel detector by Varian (mod. PS2520D, active area of 19.5 × 24.5 cm and 127 μm pixel size) were used. For the entire artwork, the *tile scanning* technique was carried out using a couple of orthogonal translation axes by Physik Instrumente in order to move the detector and obtain a field of view of about 50 × 50 cm. The CT was acquired in situ with a voltage of 80 kV, 450 μA, 2fps, 900 projections for each frame—6 frames in total—and 107 μm voxel size.

The tomography was reconstructed using the cone-beam algorithm of Feldkamp, Davis, and Kress (FDK) developed in-house^37^. Considering that this specific investigation required *tile scanning*, before the CT reconstruction the frames were stitched taking into account the opportune geometrical parameters. The 3D rendering and the image display were then performed using the two open-source software, 3D Slicer^38^ and ImageJ^39^.

The analysis is non-destructive and non-invasive, and the investigation performed did not damage the artwork in any way, as reported by Patten et al. for the CT analysis of a parchment, i.e. a more fragile material^40^. Its availability in situ also avoids any unnecessary and risky transport of the artwork.

The CT investigation does not require any specific environmental conditions, and radiation safety is guaranteed by a no-go zone based on the criteria established by a qualified expert in radiation protection. The CT of Ecce Homo painting was performed in an area that was temporarily not accessible to the public and, during the X-ray activities, the access was forbidden to everyone (including authorized operators).

### Visible and near-infrared hyper-spectral imaging

Reflectance hyper-spectral imaging (HSI) is a well-established non-invasive technique for the non-invasive analysis of polychrome surfaces^41–44^. HSI systems acquire sequences of reflectographic spectral images registered at contiguous and narrow spectral bands (with a bandwidth of a few nanometres) over an extended spectral interval, typically covering the visible (VIS), near-infrared (NIR) and short-wave infrared (SWIR) regions. The dataset acquired, called the ‘image- cube’, contains both the spectral and spatial information on the surface, so that a high-resolution reflectance spectrum is associated with each pixel of the imaged area.

The Vis–NIR-SWIR reflectance spectra enable the identification of most pigments, some preparation materials (e.g., gypsum) and selected alteration products. Pigments are usually identified through a comparative evaluation of the Vis–NIR-SWIR reflectance with reference spectral libraries. However, for certain classes of pigments, if used alone, HSI does not provide a definitive identification, and in these cases, a multimodal approach is needed. Data fusion of HSI data with MA-XRF has proven to be highly effective in providing not only an almost complete identification of pigments, but also insights into the artistic technique^45,46^. In addition, HSI data acquired in the Vis range are used to extract colour (RGB) images.

Given the large size and inherent redundancy of HSI datasets, data analysis requires the use of multiple techniques, including dimensionality-reduction methods based on statistical multivariate analysis and classification algorithms. Of these, spectral angle mapping (SAM) is a supervised method effectively used for mapping materials distributions, able to provide elaborated images highlighting the abundances and occurrences of materials, pigments and their mixtures over the painted surface. HSI data can also be processed using multivariate statistical algorithms (e.g., principal component analysis, PCA), in order to extract a few significant images which highlight elements that are not visually appreciable. The NIR-SWIR image cube is on the other hand usable to investigate underneath features, traits, and hidden details of the inner pictorial layers (such as preparatory drawings, *pentimenti*. etc.). In addition, depending on the conservation issues, spectral selection of suitable bands, as well as their recombination in false colour images, is an effective way to highlight elements that are not visually appreciable.

In the Ecce Homo painting, HSI was performed using the latest version of the IFAC-CNR HSI scanner, which is a high-performance HSI imager designed and optimised for application on valuable paintings^47^. This customised prototype provides high spatial and spectral resolution data with a minimal lighting impact on the object.

The IFAC-CNR HSI scanner is based on push-broom technology, and it includes two interchangeable spectrographic heads, based on prism-grating-prism (PGP) line-spectrographs (Specim mod. ImSpector V10E and mod. ImSpector N1E), which cover the VNIR (400-900 nm) and the SWIR (900-1700 nm) ranges, respectively. The VNIR spectrographic head uses an ORCA-ER camera (672 × 512 pixels, by Hamamatsu), whereas in the SWIR, the spectrographic head is based on a high-sensitivity InGaAs camera Xenics Xeva 1.7–640. The system operates with a spectral resolution of about 2.5 nm in the VNIR and about 10 nm in the SWIR range. Bi-telecentric optical objectives (Opto-Engineering) are coupled with the two spectrographs to guarantee the highest image quality and eliminate any geometrical distortion due to the unevenness of the support planarity. The illumination system includes a 3200 K 150-W Quartz Tungsten Halogen (QTH) lamp connected to two fibre-optic line-lights, angled at 45° to the artwork, so as to adopt a 2 × 45°/0° configuration, which is compliant with the CIE recommendations for colorimetric measurements^47^. The line-lights are fixed with the spectrographic head (VNIR or SWIR), in order to obtain a unique acquisition/illumination module that moves forward during the scanning. This module is mounted on a mechanical structure consisting of two high precision movements along an orthogonal axis on a vertical plane parallel to the painting surface. The structure allows a coverage of about 1 × 1 m area. For the Ecce Homo painting, the entire surface was acquired in one session without the need for mosaicking data.

The acquisition was performed at a speed of about 1.5 mm/sec along the vertical axis. Only a short strip (6.5 cm) of the surface is illuminated and imaged in time during acquisition, thus avoiding unnecessary light exposure on the rest of the painting. Using this set up the overall light exposure, preliminarily assessed with laboratory tests, is fully compliant with the recommended limits of light exposure in museum environments^48^. The spatial sampling was 9.2 points/mm in the SWIR and 11.4 points/mm in the VNIR. Calibration was performed by acquiring a 99% reflectance white certified Spectralon target, which was placed on a tripod next to the surface to be imaged. The wavelength calibration was performed with spectral calibration lamps (Kr, Hg, Xe, He).

The HSI measurements on Ecce Homo provided two image-cubes, in the VNIR range and in the SWIR ranges, respectively. Firstly, from the VNIR cube, the colorimetric values were calculated per image pixel, and the RGB calibrated image was reconstructed, with a spatial sampling of 11 points/mm (corresponding to a spatial resolution of about 300dpi). This RGB image was used as a reference for a comparative evaluation with other data, maps, and elaborated images in data-fusion operations. The HSI data were analysed using ENVI software^49^. Analytical methods included different approaches, such as PCA, SAM, and false colour images with spectral band selection.

### UV fluorescence

UVF imaging is a non-invasive technique routinely employed before restoration and cleaning to document and monitor the conservation procedures. Images obtained with UVF enable straightforward discrimination between certain materials that are not distinguishable under visible light, but assume a different colouration when they are lit with UV radiation. Although the fluorescence phenomenon attains only to selected substances, several pictorial materials, such as binders, varnishes, colourants, inks and, in a few cases, some inorganic pigments, exhibit fluorescence. UVF thus enables a prompt visualization of the presence of retouches, alterations in the pictorial films, non-original materials, and aged varnish layers.

The acquisitions were performed using a Nikon D600 digital camera, featuring a 24 Megapixel CCD Nikon FX array, and optical objective Nikon FX AF-D 50 mm, equipped with a Schott KV 418 filter to cut off reflected UV radiation. Two readapted Wood lamps (maximum emission wavelength at 365 nm), equipped with a DUG11 filter were symmetrically placed in the configuration 2 × 45°/0° to irradiate the target*.*

### X-ray fluorescence

X-ray fluorescence (XRF), both on single spot and scanning mode, is one of the most used in Cultural Heritage diagnostics, as it enables in situ multi-elemental, non-invasive and non-destructive analyses: no sampling or sample preparation is required, no damage to the analysed area^50–54^. The XRF technique allows the characterization of inorganic materials (often including medium- and high-Z elements), providing useful information on manufacturing techniques, dating, provenance, authenticity, and conservation state^55^.

MA-XRF measurements were performed with a portable XRF scanner developed by the INFN-CHNet network (Florence)^56–60^. The portable scanner, customised for CH diagnostics, is compact, lightweight, and easy-to-handle, with a dynamic positioning system that enables non-planar surfaces to be analysed, and a helium-flow control system for detecting atomic number elements down to sodium.

No specific environmental conditions are required, and the instrument is designed to minimize the emitted radiation, allowing for in situ measurement also in public places (e.g., museums)^56^. Moreover, the XRF scanner assures a total non-invasiveness, being equipped with an X-ray tube less powerful (4 W) than conventional tubes and energy dispersive systems (e.g., 100 s, 100 W tube-power), which has been proved secure, with no visible harm to paintings^54^.

The instrument measuring head is equipped with a Mo-anode X-ray tube (Moxtek, 40 kV maximum voltage, 0.1 mA maximum anode current) and an SDD detector (Amptek). The scanning procedure is controlled via homemade software, which controls the motion along the three-axis precision positioning stages (Physik Instrumente, 300 mm travel range in X-, 150 mm Y- and 50 mm in Z-direction), data acquisition, processing and reconstruction of the elemental distribution maps. Elemental distributions over a surface of a relatively large area undoubtedly lead to far more significant and reliable results than those obtained from multiple single-spot analyses.

The elemental maps are obtained through custom elaboration software^56^ by selecting a region of interest (ROI) in the spectrum, usually an energy interval corresponding to the characteristic X line of an element. Each pixel is assigned a grayscale level corresponding to the X-ray counts of the selected peak: white is assigned to the maximum count value, while black to the minimum. As a consequence, the same grey tone of the elemental maps obtained from different ROI of the same spectrum may correspond to different counts and/or the different colours may correspond to the same number of counts.

The experimental conditions for this campaign were: 28 kV anode voltage, 50 μA filament current, 2 mm/s scanning velocity and 1 mm of pixel size, beam diameter ~ 1 mm, no helium flow. Areas 3, 7 and 8 were acquired in different conditions and with a helium flow. Area 3 was acquired with 1 mm/s scanning velocity, 0.5 mm pixel size and 1 mm diameter beam dimension; area 7 with 2 mm/s scanning velocity, pixel size 0.5 mm and beam size 1 mm; area 8 with 1 mm/s scanning velocity, 0.25 mm pixel size and diameter of beam dimension ~ 500 µm.

## Supplementary Information

Supplementary Information.

## Data Availability

The authors of the article entitled “"Ecce Homo" by Antonello da Messina, from non-invasive investigations to data fusion and dissemination” Fauzia Albertin, Chiara Ruberto, Costanza Cucci, Marco Callieri, Marco Potenziani, Eliana Siotto, Paolo Pingi, Roberto Scopigno, Matteo Bettuzzi, Rosa Brancaccio, Maria Pia Morigi, Lisa Castelli, Francesco Taccetti, Marcello Picollo, Lorenzo Stefani, Francesca de Vita – declare hereby data availability.
