# Supplementary material for: “Ecce Homo” by Antonello da Messina, from non-invasive investigations to data fusion and dissemination
Source: Sci Rep. 2021 Aug 5;11:15868. doi: 10.1038/s41598-021-95212-2 (PMC8342594; doi:10.1038/s41598-021-95212-2)
Supplement: Supplementary file 1 — Supplementary Information. [file 41598_2021_95212_MOESM1_ESM.pdf]

“Ecce Homo” by Antonello da Messina, from non-invasive investigations to data fusion and dissemination

Fauzia Albertin[1,2,3], Chiara Ruberto[4,5*], Costanza Cucci[6*],

Marco Callieri[7], Marco Potenziani [7*], Eliana Siotto[7], Paolo Pingi[7], Roberto Scopigno[7],

Matteo Bettuzzi[2,3], Rosa Brancaccio[2,3], Maria Pia Morigi[2,3],

Lisa Castelli[4], Francesco Taccetti[4],

Marcello Picollo[6], Lorenzo Stefani[6],

Francesca de Vita [8]

[1] Enrico Fermi Historical Museum of Physics and Study and Research Center, 00184, Rome, Italy

[2] INFN - National Institute of Nuclear Physics, Bologna, 40126, Bologna, Italy

[3] Department of Physics and Astronomy “Augusto Righi”, University of Bologna, 40126, Bologna, Italy

[4] INFN - National Institute of Nuclear Physics, 50019, Sesto Fiorentino, Florence, Italy

[5] Department of Physics and Astronomy, University of Florence, 50019, Sesto Fiorentino, Florence, Italy

[6] CNR - IFAC - National Research Council, Institute of Applied Physics “Nello Carrara”, 50019, Sesto Fiorentino, Florence, Italy

[7] CNR - ISTI - National Research Council, Institute of Information Science and Technologies “Alessandro Faedo”, 56124, Pisa, Italy

[8] ALEF Conservation and Restoration Company, 43121, Parma, Italy

[*] marco.potenziani@isti.cnr.it; ruberto@fi.infn.it; c.cucci@ifac.cnr.it

Lead white and gypsum reflectance spectra

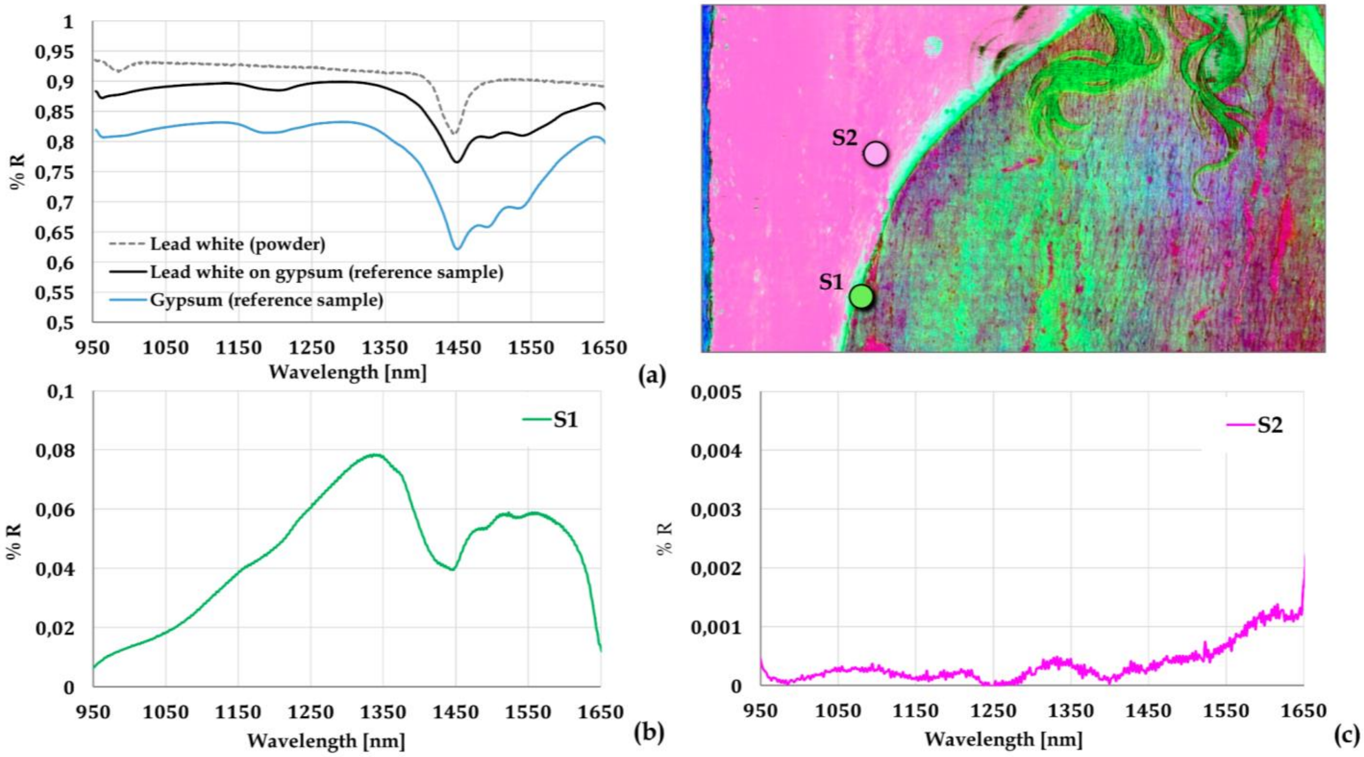

Supplementary Figure S1. Analysis of the underneath layers using hyperspectral imaging (HSI) data in the short-wave infrared range (SWIR): evidence of the presence of both lead white and gypsum a) Reflectance spectra acquired on reference materials and mock-samples[9]: the reflectance spectrum of pure lead white acquired on powder of Lead(II) carbonate basic, (grey dotted line) is reported along with the reflectance spectra acquired on reference mock-painting samples of a gypsum layer (blue continuous line) and of a lead white paint layer applied over the gypsum preparation (black continuous line). It can be observed that the pure lead white wide absorption band at 1447n overlaps with the first sub-band of the gypsum triplet (sub-bands at 1445nm, 1490nm, 1540nm, respectively). This results in the modified triplet shape when the lead white is applied over gypsum, with a typical widened and strong band at 1450nm. b) The SWIR reflectance spectrum extracted from S1, an area where the dark surface pictorial film is thinner (mapped in green). Note the typical spectral behaviour of lead-white on gypsum, with a widened band and deep band at 1450nm and two very weak sub-bands at 1490nm and 1440nm. c) The SWIR reflectance spectrum extracted from S2, as a typical area where the dark paint of the background surrounding the Christ figure is thick (mapped in pink). The reflectance signal is completely absorbed by the dark pigment on the surface.

Flesh tone XRF- spectrum

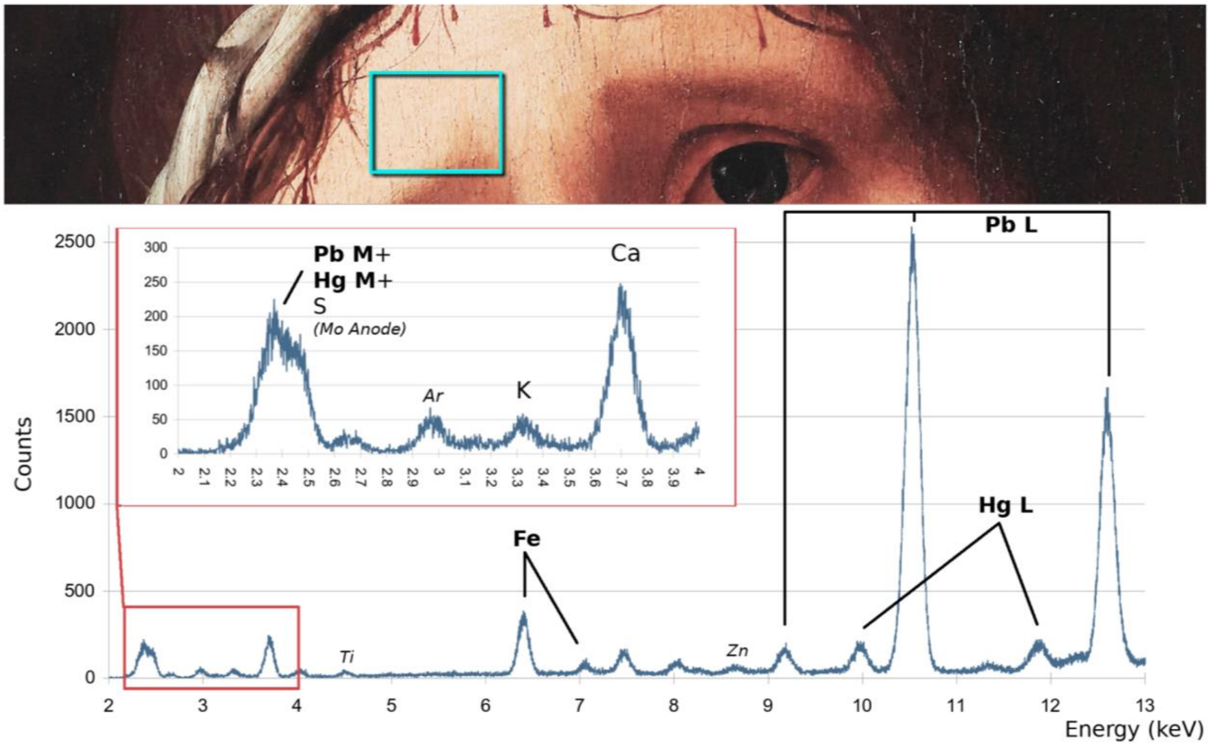

Supplementary Figure S2. XRF spectrum obtained by post-processing the MA-XRF map of the visage. The area inside the cyan square was selected in order to characterise the flesh tone, as there are very few retouches in this area (as shown in the UV image in Figure 8a). The spectrum shows the presence of Pb, Hg, and Fe, as characteristic elements of the flesh tone. The Ti and Zn traces visible in the spectrum are due to minor integrations.

Beard XRF- spectrum

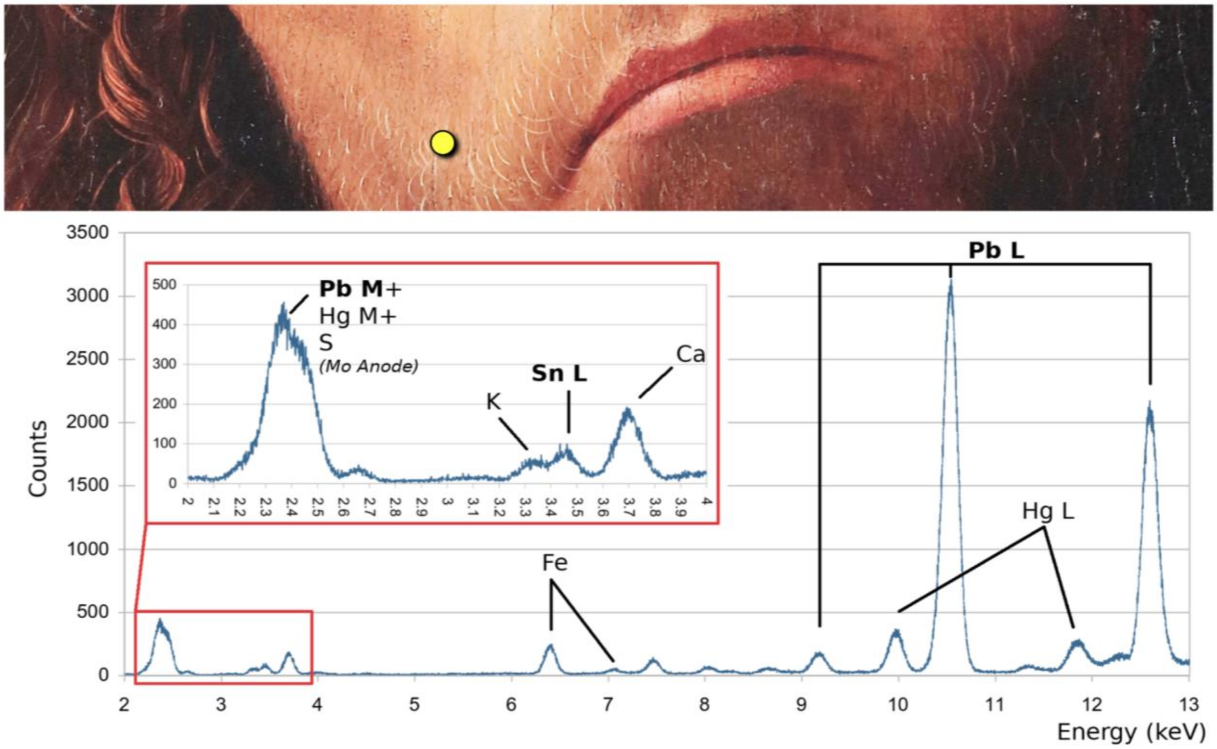

Supplementary Figure S3 The XRF spectrum acquired on the beard (with He flow) shows the presence of Sn and Pb. The measurement point is indicated by the yellow spot on the visage detail at the top of the figure. In the magnification of the low energy range of the spectrum (red square) the Sn-L line at 3.44 keV is clearly visible (see for comparison figure S2). In the spectrum, the characteristic elements of the flesh tone (Pb, Hg, Fe) are also present.

Red-lake and vermillion VNIR reflectance spectra

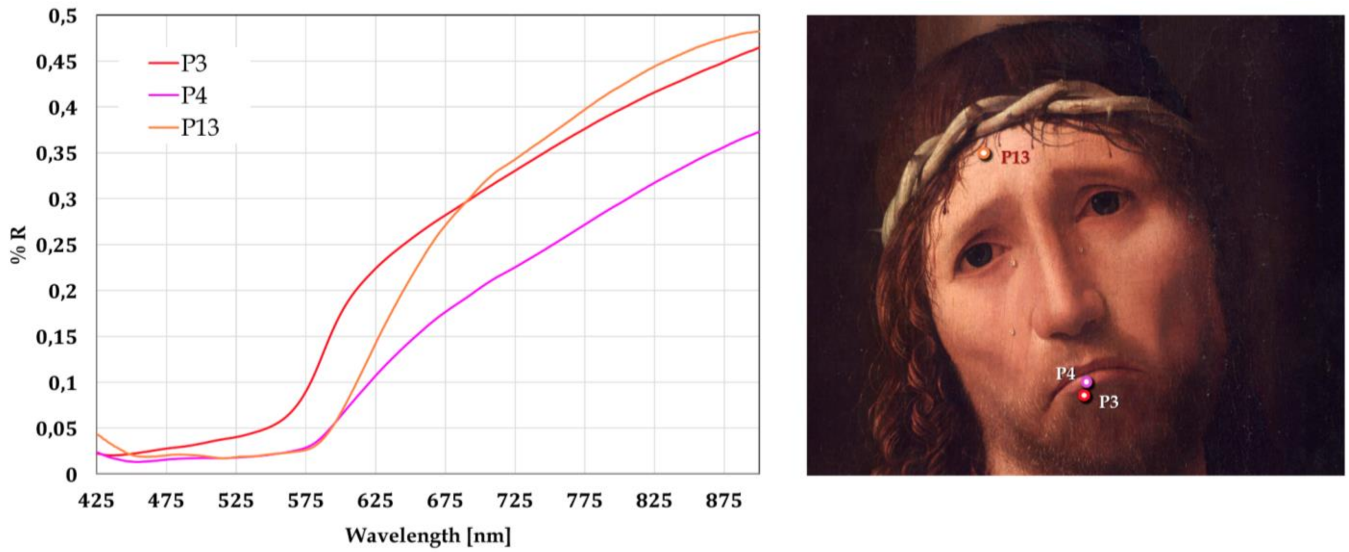

Supplementary Figure S4. VNIR spectra extracted from the HSI data in correspondence of P3, P4, and P13 points, which evidence the use of different red pigments to obtain diverse red nuances. The reflectance spectrum in P3 features the typical S-shape of vermillion, thus indicating a predominance of vermillion red; the reflectance spectrum in P4, despite its closeness to the previous one, features a different spectral behaviour, with a smother slope above 600 nm and a weak structure in sub-bands in the 450-550nm region, suggesting a possible minor presence of red-lake along. Instead, in the spectrum in P13, which corresponds to a darker red of the blood drop, the two sub-bands diagnostic of red-lake at about 515 nm and 530 nm, are much more clearly detectable thus suggesting a higher concentration of this pigment within the drop area.
